# A molecular dynamics study of membrane positioning for 7-transmembrane RGS proteins to modulate G-protein-mediated signaling in plants

**DOI:** 10.1016/j.csbj.2025.04.013

**Published:** 2025-04-11

**Authors:** Celio Cabral Oliveira, Eduardo Bassi Simoni, Mariana Abrahão Bueno Morais, Elizabeth Pacheco Batista Fontes, Pedro A. Braga dos Reis, Daisuke Urano, Alan M. Jones

**Affiliations:** a Brazilian Biorenewables National Laboratory (LNBR), Brazilian Center for Research in Energy and Materials (CNPEM), Campinas, SP 13083-970, Brazil; b Department of Biology, University of North Carolina, Chapel Hill, NC 27899, USA; c Department of Biochemistry and Molecular Biology, BIOAGRO, Universidade Federal de Viçosa, Viçosa, MG 36570-000, Brazil; d Temasek Life Sciences Laboratory, 117604, Singapore; e Department of Pharmacology, University of North Carolina, Chapel Hill, NC 27899, USA

**Keywords:** Arabidopsis, Regulator of G protein signaling (RGS), Linker domain, Heterotrimeric G protein, Molecular dynamics, Protein evolution

## Abstract

Protein phosphorylation regulates G protein signaling in plants. AtRGS1 primarily modulates AtGPA1, the canonical Gα subunit in the heterotrimeric G protein complex. AtRGS1 possesses both a seven-transmembrane (7TM) domain connected to a cytoplasmic Regulator of G Protein Signaling domain (RGS box domain) by a flexible linker region. This study presents the novel function of a highly conserved, known phosphorylation site, Ser278, within this linker region utilizing molecular dynamics (MD) simulations with *in vivo* experimental validation. We show that phosphorylation at Ser278 is crucial for establishing specific AtRGS1 interactions with AtGPA1, primarily by stabilizing the positioning and orientation of the RGS domain within the membrane. Phosphorylation at Ser278 enhances the formation of stable hydrogen bonds between phosphorylated Ser278 and conserved residues within the RGS box domain, influencing the flexibility of RGS domain mobility and thus modulating its interface to AtGPA1. Consistent with the MD simulations, *in vivo* assays demonstrated that this phosphorylation reduced the binding of AtRGS1 to AtGPA1 and conferred changes in physiology. Specifically, the non-phosphorylation mutation of Ser278 decreased both plant immune responses and AtRGS1 endocytosis evoked by the bacterial effector, flg22. MD simulations and sequence analysis of diverse plant 7TM-RGS proteins suggest conservation of this mechanism across land plants, emphasizing the critical role of this previously overlooked linker region.

## Graphical Abstract

**Figure:**
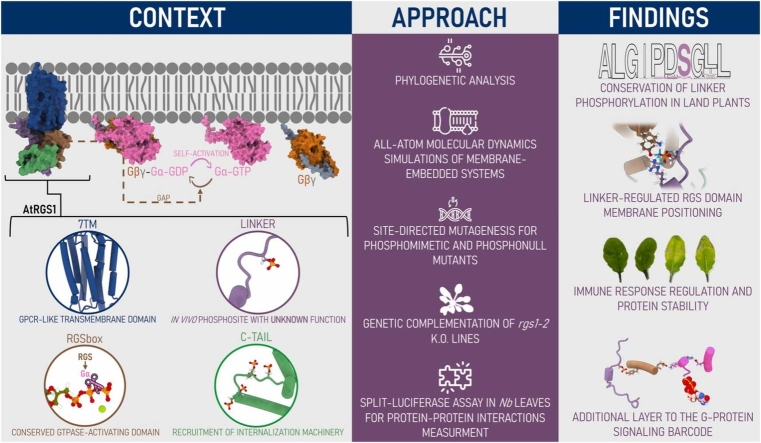

## 1 Introduction

### 1.1 The plant heterotrimeric G protein complex

Heterotrimeric G proteins are universal signaling molecules that regulate diverse cellular processes in eukaryotes. These complexes cycle between active and inactive states through GTP binding and hydrolysis by the Gα subunit, modulated by interactions with the βγ dimer [1]. Regulator of G-protein Signaling (RGS) proteins act as GTPase-activating proteins (GAPs), accelerating GTP hydrolysis to terminate signaling [2], [3]. In plants, G-protein signaling deviates from the canonical paradigm observed in animals. Specifically, while animal systems require G-protein-coupled receptors (GPCRs) for nucleotide exchange, the Gα subunit in plants spontaneously exchanges GDP for GTP. Consequently, the rate-limiting step of the GTP/GDP cycle is hydrolysis, making RGS proteins crucial for controlling signal propagation [4], [5], [6].

**Fig. 1:**
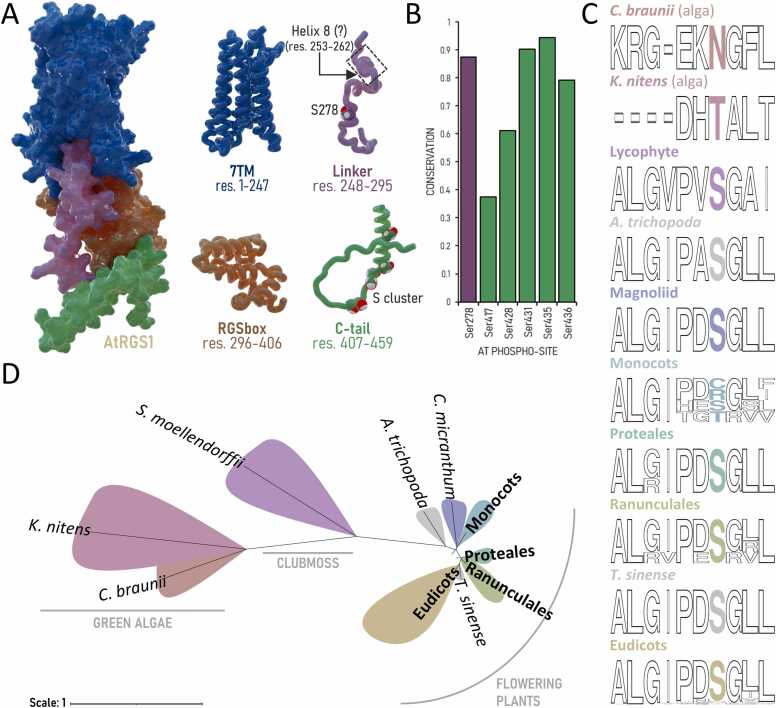
**7TM-RGS domain organization and phosphorylation sites conserved across plant species. (A)** The structural model of AtRGS1 illustrating its domain architecture: the seven-transmembrane (7TM) domain in dark blue, the flexible linker region in purple, the RGS catalytic domain in brown, and the C-terminal tail (C-tail) in green. Serine phosphorylation sites are represented as spheres. Predicted short Helix 8 is indicated with the dotted box. **(B)** Conservation of phosphorylation sites within the linker (in purple) and C-tail (in green), matched to AtRGS1 residue numbers. A bar chart shows the fractional conservation score across 72 plant species. **(C)** Multiple sequence alignment focused on the linker region across evolutionary clades from algae to eudicots. The sequence conservation was generated by WebLogo (https://weblogo.berkeley.edu/) in sequence logo representation, highlighting the S278-equivalent positions in varying colors. **(D)** A simplified phylogenetic tree mapping the evolutionary distribution of 7TM-RGS proteins. The tree illustrates the diversification of 7TM-RGS from algae to eudicots. Detailed evolutionary relationships and the full list of analyzed species are presented in Fig. S1, Appendix A.

Arabidopsis AtRGS1, a unique seven-transmembrane (7TM) protein with an RGS domain, integrates these distinct features of plant G-protein signaling. Its modular architecture (Fig. 1A), comprising a 7TM GPCR-like domain, a flexible linker, and a conserved C-terminal region (tail) rich in phosphorylation sites (C-tail), enables AtRGS1 to act as a key modulator of heterotrimeric G-protein activity [5], [7]. This structural specialization allows plants to adapt to dynamic environmental conditions [8], [9], where efficient coordination of nutrient, light, and stress signaling is essential for survival.

### 1.2 Physiological functions and phosphorylation dynamics of AtRGS1

AtRGS1 plays a central role in integrating environmental cues, such as nutrient availability and biotic stress, into intracellular signaling pathways [6], [10], [11]. For example, AtRGS1 undergoes endocytosis in response to extracellular stimuli, including D-glucose, or more likely a glucose metabolite, and pathogen-associated molecular patterns like flg22. This endocytosis is partially triggered by phosphorylation at specific residues in its cytoplasmic C-terminal regions, facilitating AtRGS1's dissociation from the G-protein complex and subsequent signal propagation [12], [13].

In plants, AtRGS1 endocytosis parallels GPCR desensitization in animals, which also relies on phosphorylation-driven internalization [14], [15], [16]. However, while animals utilize β-arrestin proteins, plants employ arrestin-like VPS26 proteins to mediate endocytosis and signal propagation [7], [13]. Additionally, AtRGS1 supports dose-duration reciprocity, enabling plants to process both transient and sustained signals—an essential property for balancing energy utilization, growth, and defense responses [8], [17].

In the absence of an experimentally-determined structure for AtRGS1 or any other 7TM-RGS proteins, recent studies have employed comparative structural homology modeling, molecular dynamics simulations, and biochemical assays to investigate plant Gα-RGS interactions. These approaches have uncovered conserved structural features and functional dynamics across diverse plant species [18]. However, these studies primarily focused on the RGS domain of 7TM-RGS proteins, often excluding the linker region, the C-terminal tail, and the transmembrane domain. These omitted regions are critical for imposing movement constraints and regulating the dynamics of the full-length AtRGS1-AtGPA1 dimer. For instance, the linker region has been shown to play an essential role in proper endoplasmic reticulum export and protein folding [19]. Despite significant progress in understanding AtRGS1 function, the role of phosphorylation at specific sites remains incompletely elucidated beyond the well-characterized C-tail serine cluster [12], [13]. Phosphorylation of Ser278—a linker residue conserved across plant species **(**Fig. 1**)**—has been detected *in vivo* [20], [21], [22] but remains underexplored. This knowledge gap may stem from the inherent challenges associated with producing and manipulating full-length membrane proteins. Traditionally regarded as structurally inert, linker regions are now recognized as dynamic regulators [23]. Based on our modeling of 7TM-RGS structures **(**Fig. 1**A)**, we hypothesize that the linker region of 7TM RGS proteins interact with the RGS box domain and the C-terminal tail in a phosphorylation-dependent manner, a mechanism conserved across plant organisms. Such interactions may modulate AtRGS1’s membrane orientation, G-protein binding affinity, and GAP activity, thereby linking structural flexibility to functional adaptability in plant signaling pathways.

### 1.3 Approach

In this study, we focused on elucidating the regulatory mechanisms underlying AtRGS1 function, with particular emphasis on linker phosphorylation-dependent modulation. We employed a genetic approach combined with biochemical assays and MD simulations **(Appendix A – Experimental Procedures)** to investigate the influence of phosphorylation of AtRGS1-Ser278 on G-protein structure, signaling and biotic stress response pathways. Additionally, we examined evolutionary constraints on AtRGS1, highlighting the significance of its conserved linker region in plant organisms. Our findings corroborate with the theory that phosphorylation patterns act as a regulatory bar code [8], [22], dynamically modulating interactions between AtRGS1 domains and controlling GAP activity.

## 2 Results and discussion

### 2.1 Flexible linker region is conserved among plant organisms

Linker domains provide flexible, non-specific connections within a protein. Lacking specific functional roles, their sequences tend to be less constrained by evolutionary pressure. This contrasts to functional molecular features such as residues for catalytic sites, protein-protein interaction surfaces, and PTMs like phosphorylation sites that remain conserved over long periods of evolutionary time due to functional constraint on evolution. Analysis of 72 plant 7TM-RGS protein sequences revealed that most phosphorylation sites identified *in vivo* [22] are highly conserved among land plants but not in algae (Fig. 1B-1C & Figure S1, Appendix A). The phosphosite Ser278 in the linker as well as adjacent residues exhibit high conservation from lycophytes to eudicots (Fig. 1C), suggesting that evolutionary constraints occur on the phosphorylation region within the linker, possibly to maintain its functional role. The origin of 7TM-RGS genes can be traced back to Streptophycean algae; however, these genes were lost multiple times (e.g., in bryophytes and some cereals) during the evolutionary transition from algae to angiosperms [24] (Fig. 1**D**). These gene loss events, combined with residue-level mutations, may have contributed to the diversification of G protein regulatory mechanisms in plants.

### 2.2 AtRGS1 structural models indicate increased stability upon Ser278 phosphorylation

To explore the structural impact of phosphorylation at Ser278, we used AlphaFold3 [25] to model AtRGS1, including both phosphorylated and non-phosphorylated variants. The top-ranked models exhibited high predicted local distance difference test (plDDT) scores for most regions (Figure S2, Appendix A), reflecting confidence in the structural predictions. As expected, the flexible regions, such as the linker and C-tail, showed medium-to-low plDDT scores due to their dynamic nature and propensity to adopt multiple conformations. Notably, introducing phosphorylation at Ser278 increased the residue’s plDDT score by 36 %, indicating enhanced structural stability at this position.

For a comprehensive evaluation of disordered regions that AlphaFold cannot model, extended MD simulations can use its structures as starting points [26], [27]. These simulations allow us to understand how phosphorylation influences the movement and stabilization of AtRGS1, providing insights into the functional significance of this modification within the linker region.

**Fig. 2:**
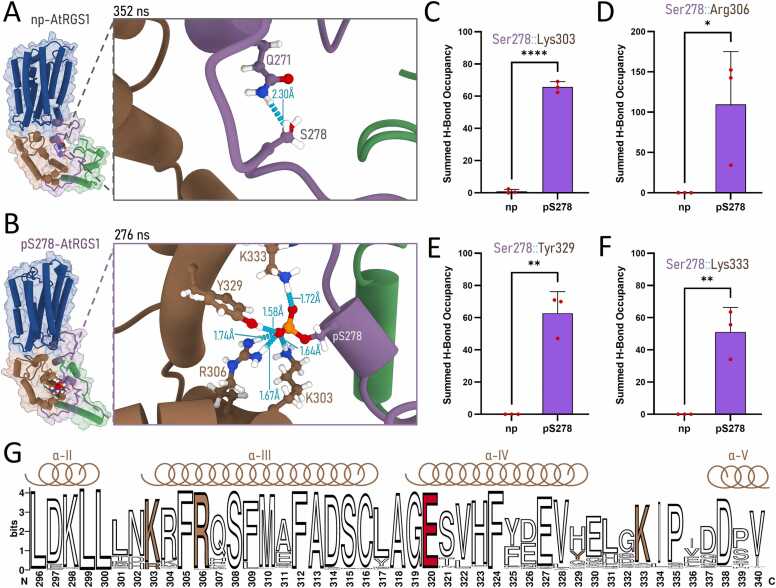
**Structural dynamics of AtRGS1 highlighted by phosphorylation at Ser278.** Centroid-frame MD simulations of AtRGS1 monomers compare **(A)** the phosphorylated Ser278 form with **(B)** the unphosphorylated form. Cartoon colors distinguish the protein’s domains: the seven-transmembrane (7TM) domain is shown in dark blue, the flexible linker region in purple, the RGS catalytic domain in brown, and the C-terminal tail (C-tail) in green. Detailed structure representations within the zoom boxes display key residues, predicted hydrogen bonds, and donor-acceptor distances indicated in cyan. **(C-F)** The average H-bond occupancies from Ser278 (gray) and phospho-Ser278 (purple) to key residues are shown for **(C)** Lys303, **(D)** Arg306, **(E)** Tyr329, and **(F)** Lys333, revealing statistically significant differences driven by phosphorylation. Unpaired t-tests; * , p < 0.05, * *, p < 0.01, * ** *, p < 0.0001**. (G)** The sequence logo for the linker-interacting RGS box region highlights the conservation of phospho-Ser278-interacting residues in brown (K303, R306, Y329, and K333) and the GAP-required residue Glu320 in red.

To probe the functionality of this linker region by phosphorylation-induced changes, we conducted all-atom MD simulations on phosphorylated and non-phosphorylated AtRGS1 models within a simulated plasma membrane environment for 400 ns**.** By analyzing representative frames (Figures S3A-S3B, Appendix A), persistent hydrogen bonds were observed between the negatively charged phospho-Ser278 and positively charged residues (Lys303, Arg306, and Lys333) and the aromatic residue Tyr329, all within the RGS box domain (Fig. 2A-2B). Comparing the H-bond occupancy during the MD simulation replicates, all interactions were completely abolished in the non-phosphorylated version of AtRGS1 (Fig. 2B-2F). Other intramolecular H-bonds observed did not show statistically distinct levels (Figures S3C-S3H, Appendix A), and RGSbox residues were mainly interacting with water molecules (Table SI, Appendix A). A similar behavior was observed during simulations of the phosphomimetic Ser-to-Glu (S278E) mutant (Figure S4, Appendix A), indicating the potential for AtRGS1 dynamics to be influenced *in vivo* by the presence of a negatively charged residue, such as a phosphoserine in the linker.

Sequence logo analysis shows that these positively charged residues are conserved across plant species. While Tyr329 is less conserved, its position exhibits prominent levels of histidine conservation, whose side chain can also serve as an H-bond donor to pS278 **(**Fig. 2**F)**. When comparing 7Tm RGS sequences among non-plant eukaryotes, we observed conservation of the residue equivalent to AtRGS1 Glu320, which is well-established for its universal role in RGS domain interaction with the G protein α subunit [4]. In contrast, the residue equivalent to Lys303 shows lower conservation, appearing in only 32.36 % of the selected RGS box domain sequences, while the Arg306 equivalent is conserved in 31.02 % of sequences. However, residues equivalent to Lys333 are highly conserved, with 81.27 % of sequences showing conservation at that position or a neighboring position, 332. At aligned position 329, we did not observe conservation of a tyrosine or histidine as found in plants, but the neighboring position 331 displays a highly conserved tyrosine residue in animals (Figure S5, Appendix A). This conservation, coupled with its proximity to the catalytic interface residue (Glu320 in AtRGS1), suggests that the pS278-interacting region is likely associated with GAP function even in fungi and animals. Furthermore, these findings support the hypothesis of a plant-specific mechanism linking GAP function to residues within the linker and RGS domain, such as Lys333 and Tyr329.

### 2.3 Phosphosite structural dynamics in 7TM-RGS proteins are evolutionarily constrained

Evolutionary analysis is also a powerful tool for assessing the functional importance of residues, and when combined with AlphaFold modeling and MD simulations, it enables the evaluation of an ensemble of residues. To investigate the conservation of phosphorylation-induced interactions between the linker and RGS box domains, we performed MD simulations of the *Selaginella moellendorffii* 7TM-RGS protein (SELML) in a comparable environment. SELML was chosen for comparison because it represents one of the most evolutionarily distant members from eudicots while exhibiting conservation of both the linker and positively-charged residues in its RGS domain (Fig. 1 & Figure S1, Appendix A). The MD simulations of SELML protein variants revealed trends consistent with those observed in AtRGS1 (Figure S6, Appendix A). Notably, the AtRGS1 Tyr329 position corresponds to an arginine in SELML (Arg322), which also forms a strong interaction with the phosphorylated equivalent (SELML-pS273) (Figure S6E, Appendix A). This suggests that the conserved interaction between the phosphosite (AtRGS1-Ser278 and SELML-Ser273) and H-bond donor residues—particularly positively charged ones—was evolutionarily constrained.

### 2.4 Ser278 phosphorylation regulates immune response in *Arabidopsis thaliana*

**Fig. 3:**
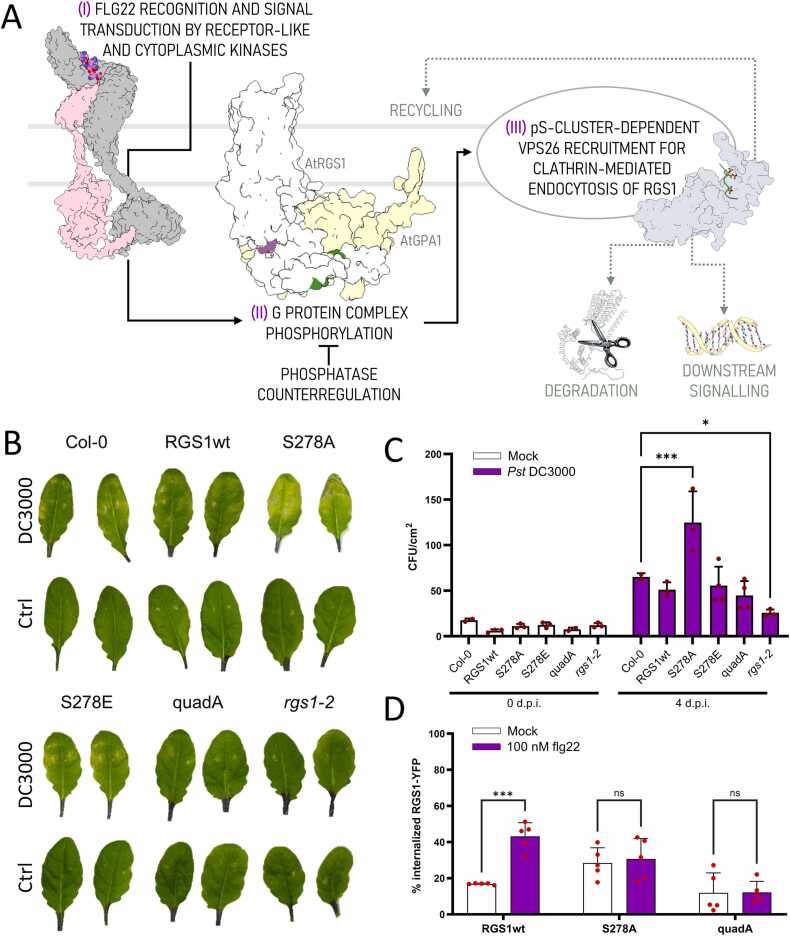
**Abolishment of Ser278 phosphorylation impaired flg22 responses in*****A. thaliana*****(A)** flg22 signaling in *Arabidopsis thaliana*. AtFLS2-AtBAK1 complex recognizes flg22, leading downstream phosphorylation and G-protein signaling cascade activation. Phosphorylated AtRGS1 dissociates from AtGPA1 and undergoes VPS26-mediated endocytosis, potentially resulting in degradation. A phosphatase complex negatively regulates this process. **(B)** Representative leaves from *A. thaliana* infected with *Pseudomonas syringae* pv. tomato DC3000 and **(C)** bacterial growth quantification in infiltrated leaves after 4 days. ANOVA, post-hoc Tukey's test comparisons to the control (Col-0); * , p < 0.05, * ** , p < 0.001. **(D)** Quantification of flg22-induced internalization of AtRGS1-YFP in dark-induced elongated hypocotyl cells. The bar graph displays the percentage of yellow fluorescence from internal regions of cells over the total fluorescence. ANOVA, post-hoc Tukey's test; * ** , p < 0.001, ns, non-significant.

flg22 is a bacterial-derived 22-aa peptide that elicits robust immune responses through the FLS2 (Flagellin-Sensing 2)/BAK1 (Brassinosteroid Insensitive 1-Associated Kinase 1)/BIK1 (Botrytis-Induced Kinase 1) complex. The G protein complex directly interacts and modulates the activity of immune responses [10], [28]. Phosphorylation at the C-terminal serine cluster of AtRGS1 induces the uncoupling of AtRGS1 from the G-protein through AtRGS1 internalization [13] (Fig. 3A). Consequently, *rgs1–2* null mutant plants exhibit a reduced pathogen colonization rate compared to wild-type plants, increased induction of flg22-related genes, loss of flg22-induced Ca^2 +^ transients, and a diminished reactive oxygen species (ROS) burst upon immune elicitation [8], [10], [13], [29], [30]. To determine the effect of Ser278 phosphorylation on AtRGS1's role in pathogen responses, we generated *rgs1–2* complemented lines expressing: 1) only the phosphomimetic version of the AtRGS1 protein (S278E), 2) the phosphonull mutant for Ser278 (S278A) or 3) the phosphonull mutations at sites Ser428, Ser431, Ser435, and Ser436 (designated quadA). Bacterial infection assays using these mutant lines showed that S278A complemented lines are more susceptible to pathogen colonization compared to a complementation line with wild type AtRGS1 (RGS1wt). As already described [10], a resistance phenotype was also noted for the non-complemented control, *rgs1–2* (Fig. 3B-3C). Similarly, flg22-induced endocytosis of AtRGS1 was abolished in both the single S278A and quadruple quadA mutants. The quantification also revealed that the basal level of internalized AtRGS1 for the S278A mutant increased, suggesting that constitutive endocytosis increased (Fig. 3D & Figure S7**, Appendix A)**.

GPCR endocytosis, and by analogy 7TM RGS protein endocytosis, is one requisite for its degradation. Therefore, having shown that the linker region of AtRGS1 is involved in its endocytosis, we examined degradation kinetics using these complemented lines. Increased stability of AtRGS1 was noted for both phosphonull mutants compared to both wildtype- and phosphomimetic-complemented lines (Figures S8-S9, Appendix A).

These findings suggest that, consistent with the previously demonstrated role of the C-tail serine cluster [12], [13], the phosphorylation of Ser278 in AtRGS1 also plays a role in its trafficking and consequently in AtGPA1 regulation and VPS26-mediated downstream signaling, which are essential for flg22 responses in *Arabidopsis thaliana* [31]. Moreover, this PTM-based modulation can be genetically encoded, underscoring its potential for engineering RGS-controlled pathways to enhance resistance in genetically modified crops.

### 2.5 Ser278 adds one more layer to the G-protein phosphorylation barcode

**Fig. 4:**
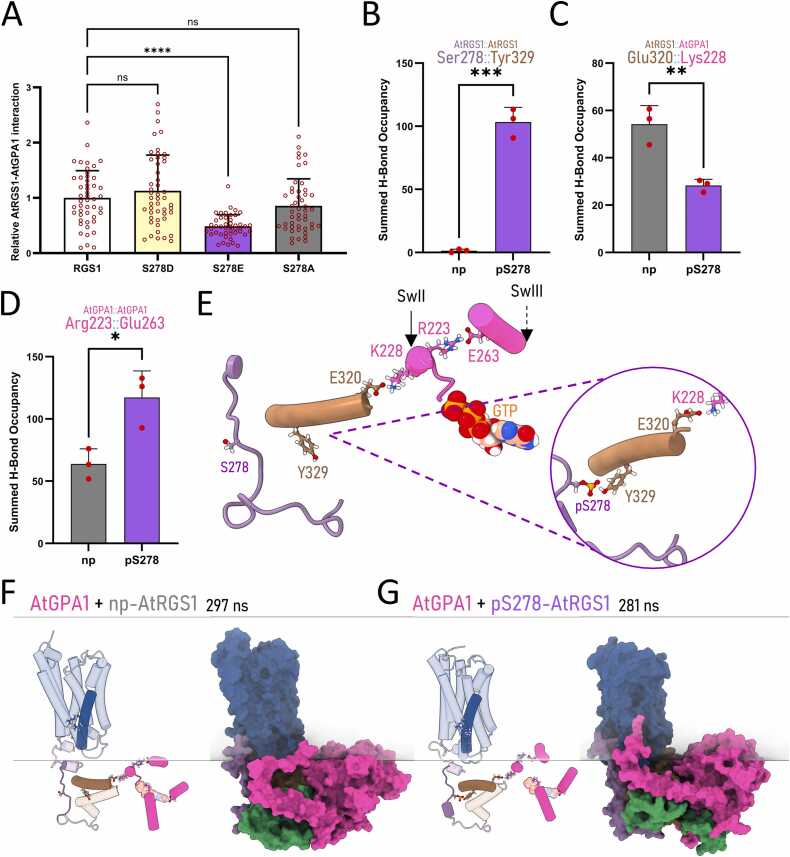
**AtGPA1 interactions coordinate with AtRGS1 linker regulation. (A)** A split-luciferase complementation assay in *N. benthamiana* leaves reveals impaired interactions between AtGPA1 and AtRGS1 S278E mutant. ANOVA, post-hoc Tukey's test comparisons to the control (RGS1, wild type); * ** *, p < 0.0001. **(B-D)** Average H-bond occupancies during 400 ns MD simulations in membrane environments are shown for AtGPA1 interactions with AtRGS1 phosphovariants: **(B)** AtRGS1 residues Ser278 and Tyr329; **(C)** AtRGS1 catalytic residue Glu320 with AtGPA1 SwII residue Lys228; **(D)** AtGPA1 SwII and SwIII residues Arg223 and Glu363. Unpaired t-tests; * , p < 0.05, * *, p < 0.01, * ** , p < 0.001**. (E)** A cartoon representation illustrates the interaction network between the RGS box domain (brown) and GPA1 residues (pink) in the np-AtRGS1/AtGPA1 dimer. The zoom box highlights the inclusion of the AtRGS1 linker region (purple) in the interaction network when Ser278 is phosphorylated. **(F-G)** PCA-selected snapshots from MD simulations depict: **(F)** the AtGPA1/np-AtRGS1 dimer at 297 ns, and **(G)** the AtGPA1/pS278-AtRGS1 dimer at 281 ns. Membrane lipids are delineated by straight lines, and the surface representations of AtRGS1 domains and AtGPA1 (pink) show regions of differential residue interactions.

Our MD simulation of AtRGS1 suggests that the phosphorylation of Ser278 stabilizes the interaction between its linker and RGS domains, potentially influencing the flexibility of the orientation of the RGS domain. Utilizing the split-luciferase assay in *Nicotiana benthamiana* plants [32], we assessed *in vivo* the effect of Ser278 phosphorylation on AtRGS1 and Gα binding at steady state. The phosphomimetic mutant S278D was included in the analysis but, as shown in Fig. 4A, it did not exhibit differences from wild-type AtRGS1. However, consistent with our previous simulations (Figure S4, Appendix A), the phosphomimetic version S278E displayed approximately 50 % reduced binding with AtGPA1 compared to both the phosphonull and non-mutated AtRGS1 proteins (Fig. 4A). Notably, although S278E cannot fully replicate the comprehensive hydrogen bonding provided by phosphoserine (Figure S4, Appendix A), its longer side chain still preserves the negative charge and key proximity to target residues in the RGS domain. Conversely, S278D exhibits a shorter side chain that diverges further from phosphoserine’s bulk and electrostatics, which likely explains why no difference was detected in the split-luciferase assay. This rationale also underscores our focus on S278E for more detailed functional analyses.

So far, we examined the phosphorylation “barcode” of AtRGS1 on interaction with AtGPA1, but AtGPA1 is also phosphorylated [33]. Specifically, the phosphorylation of Tyr166 located near the nucleotide-binding pocket of AtGPA1 dramatically changes how AtRGS1 interacts with its substrate AtGPA1 in a guanine-nucleotide-dependent mechanism called tyrosine phosphorylation switching [34]. Tyr166, likely in coordination with other N-terminal residues, is essential for BAK1-mediated phosphorylation of AtGPA1. The phosphomimetic mutation AtGPA1-Y166E also reduces AtRGS1 interaction and GTPase activity [34], [35]. Consequently, we investigated the binding strength of the AtGPA1 Y166E phosphomimetic mutant to AtRGS1 phosphomimetic (S278E) and phosphonull (S278A) mutants at Ser278. Interestingly, the S278E mutant exhibited binding strength comparable to wild-type AtRGS1, whereas the phosphonull mutant (S278A) demonstrated a significant reduction in interaction with the Y166E mutant (Figure S10, Appendix A). These results align with the four-state model theory [8], suggesting that G-protein regulation through GAP function involves coordinated phosphorylation events on several AtRGS1 regions and on its substrate, AtGPA1.

We performed 400-ns MD simulations to investigate the impact of different phosphorylation states of the AtRGS1 linker when bound to the AtGPA1-GTP complex. Consistent with our monomer MD simulations, the RGS box remained strongly bound to the phosphorylated linker throughout the simulations, primarily through hydrogen bonds with the RGS box residue Tyr329 (Fig. 4B). Furthermore, consistent with the split-luciferase assay results, Ser278 phosphorylation significantly influenced the interaction frequency between AtRGS1 and AtGPA1. This effect was highlighted by the markedly reduced H-bond occupancy of AtRGS1 Glu320 with AtGPA1 Lys228 in simulations involving pS278 (Fig. 4C). Lys228 resides in the Switch II (SwII) region of AtGPA1, one of the three dynamic switch regions designated SwI, SwII, and SwIII that undergo conformational changes during GTP binding and hydrolysis to regulate interactions with effectors and signaling proteins [34]. Consistently, Ser278 phosphorylation significantly increased the H-bond occupancy between AtGPA1 Arg223, located within the SwII region, and Glu263, which lies near the SwIII loop (Fig. 4D-4E). A similar trend was observed in GTP binding dynamics, where the tri-phosphate-binding residue Lys51 showed reduced binding in the pS278-containing dimer, while AtGPA1 Asp290, which interacts with a nitrogen group in GTP, exhibited decreased sidechain engagement in the phosphorylated dimer (Figures S11D-S11E, Appendix A). These AtGPA1 regulatory regions closely align with the phosphorylation site Tyr166, essential for nucleotide state switching (Figure S11F, Appendix A). By analyzing representative frames of the dimer simulations and the average residue correlations of AtRGS1 Ser278 with all other protein residues, we identified a sidechain interaction network that directly connects the AtRGS1 linker region to the nucleotide pocket of AtGPA1 when the linker is phosphorylated (Fig. 4E & Figure S11, Appendix A).

### 2.6 Phosphorylation of AtRGS1 linker regulates Helix 8 position and membrane interactions

Importantly, in animals, several PTM-based regulatory processes influence internalization and functional events associated with protein–plasma membrane dynamics. For example, GPCRs can undergo palmitoylation at Helix 8, which significantly affects signaling pathways [36]. Helix 8 also plays a critical role in modulating membrane curvature to facilitate endocytosis activation [37]. In both animals, yeast, and plants, lipidation of Gα and/or the Gβγ dimer serves as a key regulator of signaling [38], [39], [40]. In plants, deletion of the N-terminal helix of AtGPA1 has been shown to markedly alter its nucleotide affinity and complex stability [40], [41]. Consistently, our simulations revealed notable changes in the transmembrane and anchored regions of both AtRGS1 and AtGPA1. Specifically, a significant change in H-bond occupancy was observed for AtRGS1 residues Arg104 and Gln107, located in Helix 3, which directly interact with the predicted Helix 8 of AtRGS1 (Figure S11C, Appendix A).

By aligning and demarcating the plasma membrane region in the centroid frames of our MD systems, we observed that the SwII and SwIII regions of AtGPA1 were displaced toward the membrane when AtRGS1-Ser278 was phosphorylated. Interestingly, the N-terminal helix of AtGPA1 was found to directly interact with the C-terminal region of AtRGS1 (Figure S11I, Appendix A). This interaction, alongside the anchoring of lipidated residues on AtGPA1, appears to be modulated by the phosphorylation state of the AtRGS1 linker. Additionally, we noted that the predicted Helix 8 becomes more deeply buried within the 7TM pocket in the AtGPA1/AtRGS1-pS278 system (Fig. 4F–4 G). This observation was further supported by Solvent Accessible Surface Area (SASA) measurements across the full simulation and its replicates, which revealed a consistently lower SASA for the phosphorylated dimer system (Figure S11J, Appendix A). Finally, the spatial proximity of the C-terminal serine cluster (S-cluster) in AtRGS1 to the N-terminal phosphosites of AtGPA1 suggests a critical role for these phosphorylation events in facilitating dimer uncoupling during stress responses and VPS recruitment. Furthermore, the differential movement of the predicted Helix 8 may highlight the importance of this region, akin to its role in GPCRs, and could provide insights that broaden our understanding of plant G-protein signaling.

## 3 Conclusions

In summary, this study identifies Ser278 phosphorylation in the linker region of AtRGS1 as a regulator of G-protein signaling in plants. This modification stabilizes the RGS domain orientation at the plasma membrane by promoting hydrogen bonding with residues in the RGS box, influencing its interaction with the Gα subunit, AtGPA1. Molecular dynamics simulations indicated that phosphorylation restricts RGS domain flexibility, altering the spatial conformation of the AtRGS1–AtGPA1 complex. Experimental validation confirmed that phosphomimetic and phosphonull mutations at Ser278 affect AtRGS1–Gα binding, flg22-induced endocytosis, and immune responses, establishing a link between structural regulation and physiological output.

The high conservation of Ser278 and its interacting residues across land plants supports its functional significance. In the lycophyte Selaginella, the homologous mechanism constrains RGS domain positioning similarly, suggesting evolutionary retention of this regulatory strategy. Given the limited number of G-protein components in plants, such conserved phosphoregulation likely enhances signaling distinction without requiring expanded protein repertoires.

These findings position Ser278 within a broader phosphorylation-based regulatory system. Together with C-terminal tail modifications on AtRGS1 and known phosphosites on AtGPA1 [12], [20], [22], [33], [34], [35], [42], [43], Ser278 contributes to a combinatorial "barcode" that governs interaction dynamics, membrane engagement, and signaling specificity. Rather than acting as a binary switch, this site integrates with other post-translational modifications to finely tune G-protein activity in response to cellular context.

In this present work, we exemplify how deep learning-based predictions and all-atom MD simulations—when embedded in realistic environments—can yield testable mechanistic hypotheses. The alignment between in silico and in vivo results shows the power of integrating computational modeling with functional assays to dissect the behavior of structurally complex proteins, as transmembrane regulators.

Functionally, our insights, combined with the known parts of this “barcode”, may be used beyond model plants. While the present work focuses on biotic stress signaling, G-protein pathways are also known to mediate plant responses to abiotic cues such as drought, salinity, and temperature [44], [45], [46], [47], [48], [49]. The regulatory mechanism uncovered here may similarly operate under such conditions, raising the possibility that linker phosphorylation in 7TM-RGS proteins plays a broader role in environmental adaptation. Therefore, by modulating the phosphorylation of Ser278 or its docking interface, it may be possible to engineer crops with modified RGS–Gα coupling strength. This precise adjustment could recalibrate growth-defense trade-offs [45], thereby enhancing immunity without incurring fitness costs.

Finally, this work advances our understanding of how plants achieve regulatory complexity with limited signaling components and highlights a new opportunity for crop improvement through precision modulation of signaling dynamics.

## CRediT authorship contribution statement

**Pedro A. Braga dos Reis:** Writing – review & editing, Supervision, Methodology, Funding acquisition. **Daisuke Urano:** Writing – review & editing, Supervision, Methodology, Conceptualization. **Alan M. Jones:** Writing – review & editing, Resources, Methodology, Funding acquisition, Conceptualization. **Celio Cabral Oliveira:** Writing – review & editing, Writing – original draft, Visualization, Validation, Methodology, Investigation, Formal analysis, Data curation, Conceptualization. **Eduardo Bassi Simoni:** Writing – review & editing, Investigation, Formal analysis. **Mariana Abrahão Bueno Morais:** Writing – review & editing, Formal analysis, Investigation. **Elizabeth Pacheco Batista Fontes:** Writing – review & editing, Supervision, Funding acquisition.

## Funding and additional information

This work was partially supported by the Brazilian Coordination for the Improvement of Higher Education Personnel (CAPES, Financing Code 001) with the scope of the Capes-PrInt Program to CCO and EBS; by grants from The Research Support Foundation of the State of Minas Gerais (FAPEMIG, APQ-01357-22) and research fellowships from The Brazilian National Council for Scientific and Technological Development (CNPq, 308209/2022-2) to PABR; by grants from FAPEMIG (RED-00205-22) and CNPq (406440/2022-0) to EPBF; and by grants from the 10.13039/100000057National Institute of General Medical Sciences (NIGMS, GM065989) and from the 10.13039/100000001National Science Foundation (NSF, MCB-1713880) to AMJ.

## Declaration of Generative AI and AI-assisted technologies in the writing process

During the preparation of this work, the author(s) used GPT-4 (OpenAI) for grammar and clarity improvement of the text, as well as for processing large table datasets. After using this tool/service, the authors reviewed and edited the content as needed and take full responsibility for the content of the publication.

## Declaration of Competing Interest

The authors declare that they have no known competing financial interests or personal relationships that could have appeared to influence the work reported in this paper
