# Supplementary material for: A molecular dynamics study of membrane positioning for 7-transmembrane RGS proteins to modulate G-protein-mediated signaling in plants
Source: Comput Struct Biotechnol J. 2025 Apr 11;27:1529–37. doi: 10.1016/j.csbj.2025.04.013 (PMC12017998; doi:10.1016/j.csbj.2025.04.013)
Supplement: Supplementary file 1 — Supplementary material [file mmc1.docx]

**Appendix A – Supplementary Data**

A molecular dynamics study of membrane positioning for 7-transmembrane RGS proteins to modulate G-protein-mediated signaling in plants

Celio Cabral Oliveira^1,2,3*^, Eduardo Bassi Simoni^2,3^, Mariana Abrahão Bueno Morais^1^, Elizabeth Pacheco Batista Fontes³, Pedro Agusta Braga dos Reis^3^, Daisuke Urano^4^, Alan M. Jones^2,5^

^1^Brazilian Biorenewables National Laboratory (LNBR), Brazilian Center for Research in Energy and Materials (CNPEM), Campinas, SP, Brazil 13083-970

^2^Department of Biology, University of North Carolina, Chapel Hill, NC, USA 27899

^3^ Departamento de Bioquímica e Biologia Molecular, BIOAGRO, Universidade Federal de Viçosa, Viçosa, MG, Brazil 36570-000

^4^Temasek Life Sciences Laboratory, Singapore 117604

^5^Department of Pharmacology, University of North Carolina, Chapel Hill, NC, USA 27899

*To whom correspondence is addressed: celio.oliveira@lnbr.cnpem.br

EXPERIMENTAL PROCEDURES

Sequence alignment and phylogenetic analysis

We manually curated 7TM-RGS sequences from diverse plants covering streptophyte algae to flowering plants: A0A1Y1HMV6_KLENI (*Klebsormidium nitens*), A0A443Q1P9_9MAGN (*Cinnamomum micranthum*), A0A4U6VTJ9_SETVI (*Setaria viridis*), D8SMS6_SELML (*Selaginella moellendorffii)*, RGS1_ARATH (*Arabidopsis thaliana*), and V5SQ35_CHABU (*Chara braunii*). These initial sequences were blasted against the UniProtKB and Swissprot databases with the e-value threshold of 0.00001. The search yielded 375 sequences that were aligned using MAFFT version 7 [1]. Sequences were pre-processed by removing sequences containing less than 400 or more than 1200 amino acid residues and redundant sequences (>95% similarity). The remaining 159 sequences were realigned, followed by the removal of gapped positions containing ≥75% gaps and highly gaped sequences. The final alignment (72 sequences, 465 positions) was submitted to phylogenetic tree reconstruction using the auto-chosen substitution model “Q.plant+I+G4” and 100 bootstrap iterations in IQ-Tree version 2.2.2.7 [2]. Tree representation was made using the Interactive Tree of Life tool (iTOL) [3].

For the RGS box domain analyses within organisms that do not contain the Linker-RGS architecture, protein sequences containing the RGS domain (IPR016137), excluding Viridiplantae, were obtained from UniProtKB, totaling 61,930 sequences. These sequences were clustered using CD-HIT [4] with a 90% similarity cutoff, resulting in 20,795 sequences. The sequences were then aligned to the AtRGS1 RGS domain (residues 296 to 406) using MAFFT version 7 [1]. An attempt was also made to align the same set to the AtRGS1 linker region (residues 248 to 295), but no sequence aligned, confirming the absence of a plant-like linker region in this set. From the RGS box domain alignment, non-aligned columns were removed, and redundancy was further reduced using a 90% similarity cutoff again, resulting in a final set of 2,393 sequences. These sequences were used to create the sequence logo via the WebLogo server [5]. For the positive residues of interest, conservation percentage was calculated from this set by identifying the presence of lysine or arginine at each of the three analyzed positions (AtRGS1 Lys303, Arg306, and Lys333).

Molecular dynamics simulations and analysis

Individual chain and dimeric models were created using the AlphaFold3 algorithm [6]. For the AtRGS1-pS278- or SELML-pS273-containing systems, phosphorylation was added prior to modeling. GTP and Mg²⁺ were incorporated into all dimeric models containing AtGPA1. The initial methionine of AtGPA1 was removed from the models, and lipidation of its N-terminal residues Gly2 and Cys5, through myristoylation and palmitoylation respectively, was included to simulate a closer approximation to the *in vivo* environment [7]. Additionally, approximately 150 molecules of palmitic acid, myristic acid, and oleic acid were added during modeling, leveraging the AlphaFold3 ligand database to equilibrate the hydrophobic regions of the protein. Five models were generated, and the top-ranking models were selected based on predicted local distance difference test (pLDDT) scores for monomers and interfacial predicted template modeling (ipTM) scores for dimers [6,8].

A membrane-based system was constructed using the CHARMM-GUI membrane builder tool [9], with PPM3.0 [10] employed to accurately position the hydrophobic regions within a synthetic plasma membrane. The membrane composition was set to approximately 5:5:1 DUPC:SITO:CER1, where DUPC is 1,2-dilinoleoyl-sn-glycero-3-phosphocholine, SITO is β-sitosterol, and CER1 is ceramide D181/160, closely mimicking the plant plasma membrane composition [11]. A neutral pH environment (pH = 7) was used, with side-chain protonation, including for histidine residues, assigned by the CHARMM-GUI built-in tool. Salt was added as NaCl at a 150 mM concentration using the Monte Carlo method (11).

All systems underwent extended minimization as recommended by CHARMM-GUI, followed by equilibration in the isothermal-isobaric (NPT) ensemble using the V-rescale thermostat for temperature control and the C-rescale barostat with semi-isotropic coupling for pressure regulation, at 303.15 K [12]. Production simulations of 400 ns were performed for all systems using GROMACS [13] 2022, with each system run in three independent replicates. Simulations employed hydrogen mass repartitioning to enable a 4-fs time step [14]. Detailed system parameters, including residue protonation states, system sizes, and the number of ions added, are provided in **Table SII.**

The production simulation frames were concatenated and aligned using the protein chain atoms as a reference. Principal Component Analysis (PCA) was performed using the MD-task tool [15], utilizing the atomic coordinates of all protein atoms or the combined coordinates of protein atoms, Mg²⁺, and GTP in dimeric simulations. Hydrogen bonds (H-bonds) were identified in the centroid frames based on a stringent distance cut-off of 3.0 Å and an angle cut-off of 20°. H-bond occupancy was quantified using the HBonds plugin version 1.2 in VMD [16], which defines occupancy as the cumulative percentage of simulation time during which specific hydrogen bonds are formed between residue pairs. For residue pairs forming multiple hydrogen bonds, the plugin automatically aggregates their occupancies, resulting in total values that can exceed 100%. Occupancy frequencies were calculated for each independent replicate, and differences among phosphovariants were assessed by performing multiple unpaired t-tests in GraphPad Prism 7.

For AtGPA1-AtRGS1 simulations, Dynamic Cross-Correlation (DCC) analyses of Cα atom trajectories were also performed using the MD-task tool [15] to investigate inter-residue motion correlations. Solvent Accessible Surface Area (SASA) for AtRGS1 Helix 8 was calculated by averaging the accessibility of residues 253 to 262 across all frames and replicates using the FreeSASA tool [17]. Structural representations and interaction visualizations were generated using UCSF ChimeraX software [18].

Cloning and site-directed mutagenesis

HiBiT-tagged AtRGS1 entry clones were generated by inserting the short coding sequence at the reverse primer and inserted in the pENTR/D-TOPO vector (Invitrogen). For site-directed mutagenesis, Q5® High-Fidelity DNA Polymerase (New England Biolabs) was used for end-to-end amplification of the entry vector. Mutagenesis oligonucleotides were designed for single or multiple codon modification and a free phosphate group was added to the 5’ end of each primer. The linear vector was then ligated using the T4 DNA Ligase enzyme (Invitrogen). New clones were generated by transforming into *E. coli* DH5α and confirmed by sequencing. Cloned genes were subjected to LR Clonase II (Invitrogen) reaction and transferred to the plant expression vectors pEarleyGate 101 and pCAMBIA-NLuc. Clones from AtGPA1 on pCAMBIA-CLuc vectors were previously described [19].

Plant growth and transformation

Phosphomimetic and phosphonull versions of AtRGS1 on pEarleyGate 101 plasmid were transferred to *Agrobacterium tumefaciens* cells and transformed into *rgs1-2* plants by the floral dip method [20]. Single-insertion homozygous lines were selected by BASTA resistance and its related survival rate.

Arabidopsis plants were maintained in a growth chamber under short-day conditions (21ºC, 8h/light, 16h/dark). Seedlings for western blot analysis were germinated on ¼ strength liquid Murashige and Skoog (MS) medium and grown for 7 days under low constant light conditions. For confocal microscopy, seedlings were placed on ¼ strength liquid MS and etiolated under dark conditions [21]. *Nicotiana benthamiana* plants were germinated on soil and kept in a half-day photoperiod (25ºC, 12h/day, 22ºC, 12h/night) for 5 weeks before infiltration. Dark treatment was applied for 24 hours after infiltration.

Split-luciferase complementation assay

CLuc-tagged and HiBiT-NLuc-tagged constructs were transformed into *Agrobacterium tumefaciens* GV3101 cells and subsequently co-infiltrated into the leaves of 5-week-old *Nicotiana benthamiana* plants. Leaf disks were harvested from each biological replicate two days post-infiltration, with one disk placed per well in a 96-well plate containing either 1 mM D-Luciferin or HiBiT reaction mix (Promega). The reaction was incubated in the dark for 10 minutes, after which bioluminescence intensity was measured at 570 nm [22].

A control group of wild-type AtRGS1 was included on every plate to ensure consistent normalization across experiments. Luciferase activity for each leaf was first normalized to the average HiBiT expression level, then further normalized to the wild-type AtRGS1 control on the same plate. Each plate contained at least 12 biological replicates, and the entire experiment was repeated a minimum of four times. All normalized data were combined and analyzed using GraphPad Prism 7, where a one-way ANOVA followed by Tukey’s multiple comparisons test was performed to assess differences in luciferase activity between the mutants and the wild-type control.

AtRGS1 stability assessment

Seven-day-old seedlings from AtRGS1^WT^, AtRGS1^S278E^, AtRGS1^S278A^, and AtRGS1^S428/431/435/436A^ (quadA) lines were exposed to 100 nM flg22 and 200 μM cycloheximide over a 6-hour or 12-hour time course under constant light and agitation. Total protein was extracted using a specialized RGS1 extraction buffer containing 50 mM Tris-HCl (pH 7.5), 10% glycerol, 0.5% Triton X-100, 1.5 mM MgCl₂, 1 mM EDTA, 150 mM NaCl, 1 mM phenylmethane sulfonyl fluoride (PMSF), 1× Roche protease inhibitor cocktail, and 10 mM NaF.

Extracted proteins were resolved by SDS-PAGE on 12% acrylamide gels, and AtRGS1-YFP was detected directly in-gel using the Amersham™ Typhoon™ 5 scanner. Detection was performed with a Cy2 (525BP20) filter, using an excitation peak of 488 nm, an emission peak of 525 nm, a pixel size of 50 μm, and a photomultiplier tube (PMT) voltage set to 700 V. Following imaging, the gel was stained with Coomassie Brilliant Blue R-250, and the bands corresponding to the RuBisCO large subunit (rbcL) were used as a loading control.

flg22-induced internalization of AtRGS1

To avoid artifacts from overexpression, only plants expressing YFP-tagged AtRGS1^WT^ (112), AtRGS1^S278A^ (#345), or AtRGS1^S428/431/435/436A^ (quadA, #290) at wild-type expression levels were used in this study. Seeds were exposed to light for approximately 6 hours, and after 3 days of germination under dark conditions, etiolated seedlings were treated with either water (control) or 100 nM flg22 for 15 minutes.

Single, fully elongated hypocotyl cells were imaged using a Zeiss LSM880 confocal microscope equipped with a C-Apochromat 40x/1.2NA water immersion objective. YFP fluorescence was captured from multiple Z-layers to ensure accurate representation of the signal. Image processing and quantification were performed as previously described [19].

Bacterial infection assay

*Pseudomonas syringae pv. tomato (Pst)*, DC3000 strain, was cultured overnight at 28°C in LB medium containing 50 μg/mL rifampicin. Bacteria were harvested by centrifugation, washed, and adjusted to the desired density (OD 10^-4^) with 10 mM MgCl2. Leaves of Col-0, AtRGS1^WT^, AtRGS1^S278E^, AtRGS1^S278A^, AtRGS1^S428/431/435/436A^ (quadA) and *rgs1-2* plants at 4-week-old were infiltrated with the bacterial suspension using a 1-mL needleless syringe. Subsequently, leaves were collected to measure bacterial growth. Six leaf discs, separated as three repeats, were ground in 1mL H_2_O, and serial dilutions were plated onto TSA medium (1% Bacto tryptone, 1% sucrose, 0.1% glutamic acid, 1.5% agar) with the appropriate antibiotics. Bacterial colony forming-units were counted after a 4-day incubation at 28°C [23].

SUPPLEMENTARY FIGURES

Supplementary Figure 1. Phylogenetic analysis of 7TM-RGS proteins and conservation of phosphosites among plant 7TM RGS1 proteins


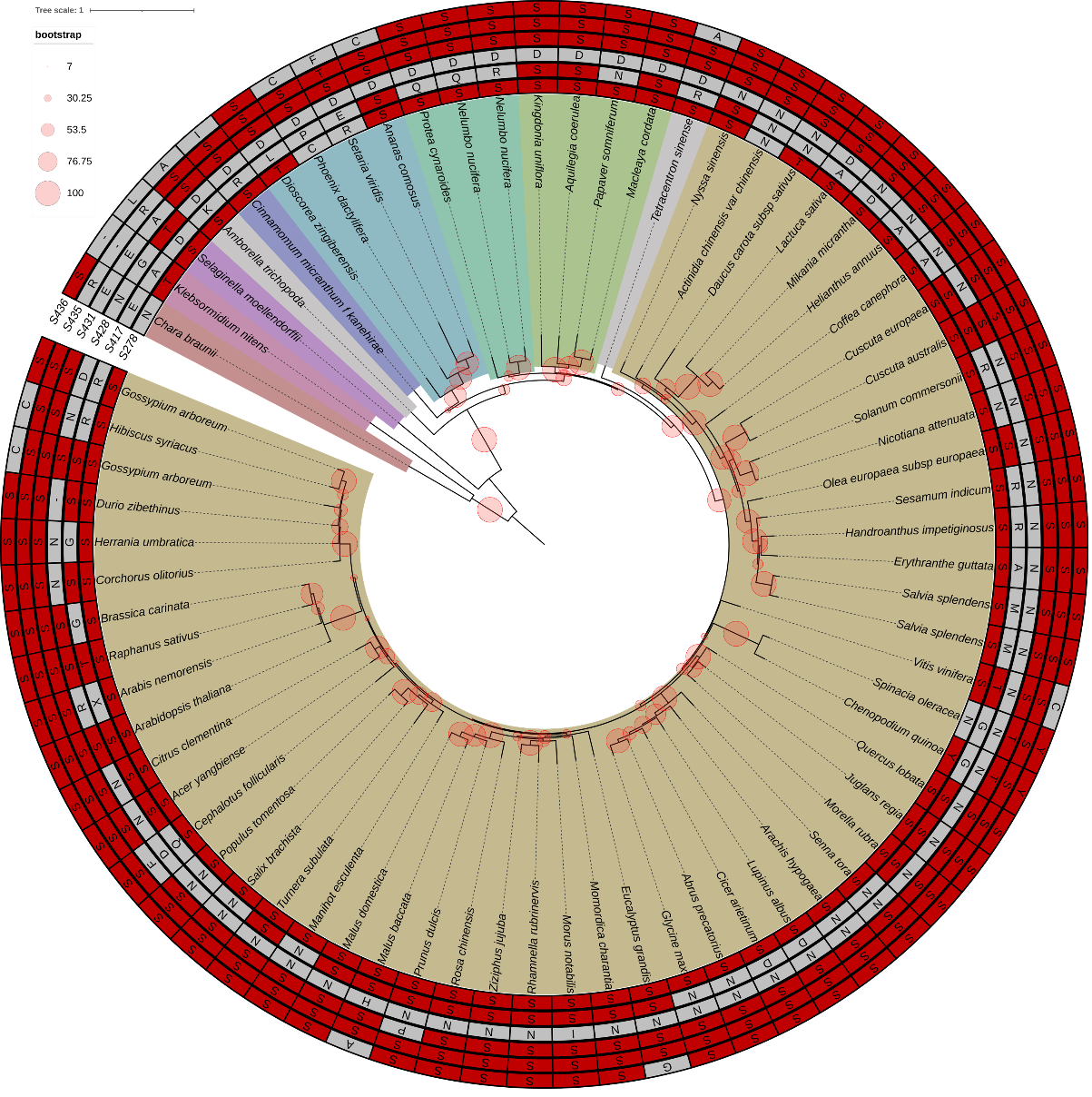


The tree illustrates the phylogenetic relationships among 7TM-RGS proteins within plant species. Transparent red circles indicate branch confidence levels derived from bootstrap analysis. Residues in the outer rings depict phosphosites identified in the linker or C-tail regions of AtRGS1. Conserved and non-conserved residues are highlighted with red and grey backgrounds, respectively, across different species, including algae (Dusty Rose & Mauve Pink), club mosses (Lavender Purple), and flowering plants including basal angiosperms (Pale Silver), magnoliids (Slate Blue), monocots (Soft Teal), proteales (Sage Green), ranunculales (Light Khaki), and eudicots (Warm Tan). A simplified tree is presented in **Figure 1D**.

Supplementary Figure 2. AlphaFold3-generated protein models used in this study

**
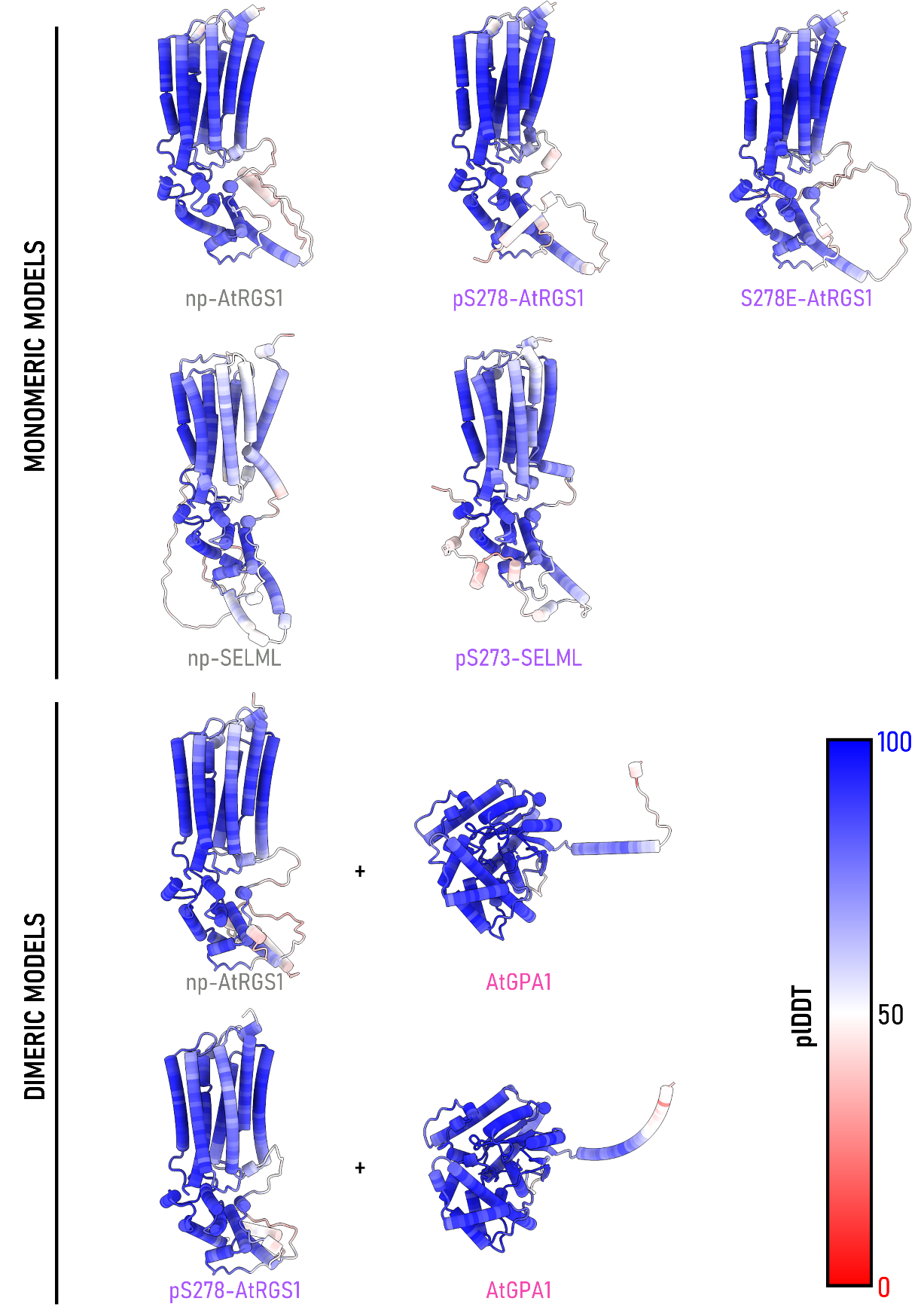
**

Top-ranked models generated using AlphaFold3 for monomeric proteins (AtRGS1 and SELML) and the AtRGS1/AtGPA1 dimer are shown. The cartoon representations are colored according to the pLDDT score, ranging from low (red) to high local confidence (blue) for the predictions.

Supplementary Figure 3. All-atom MD simulations on phosphorylated and non-phosphorylated AtRGS1 models.

**
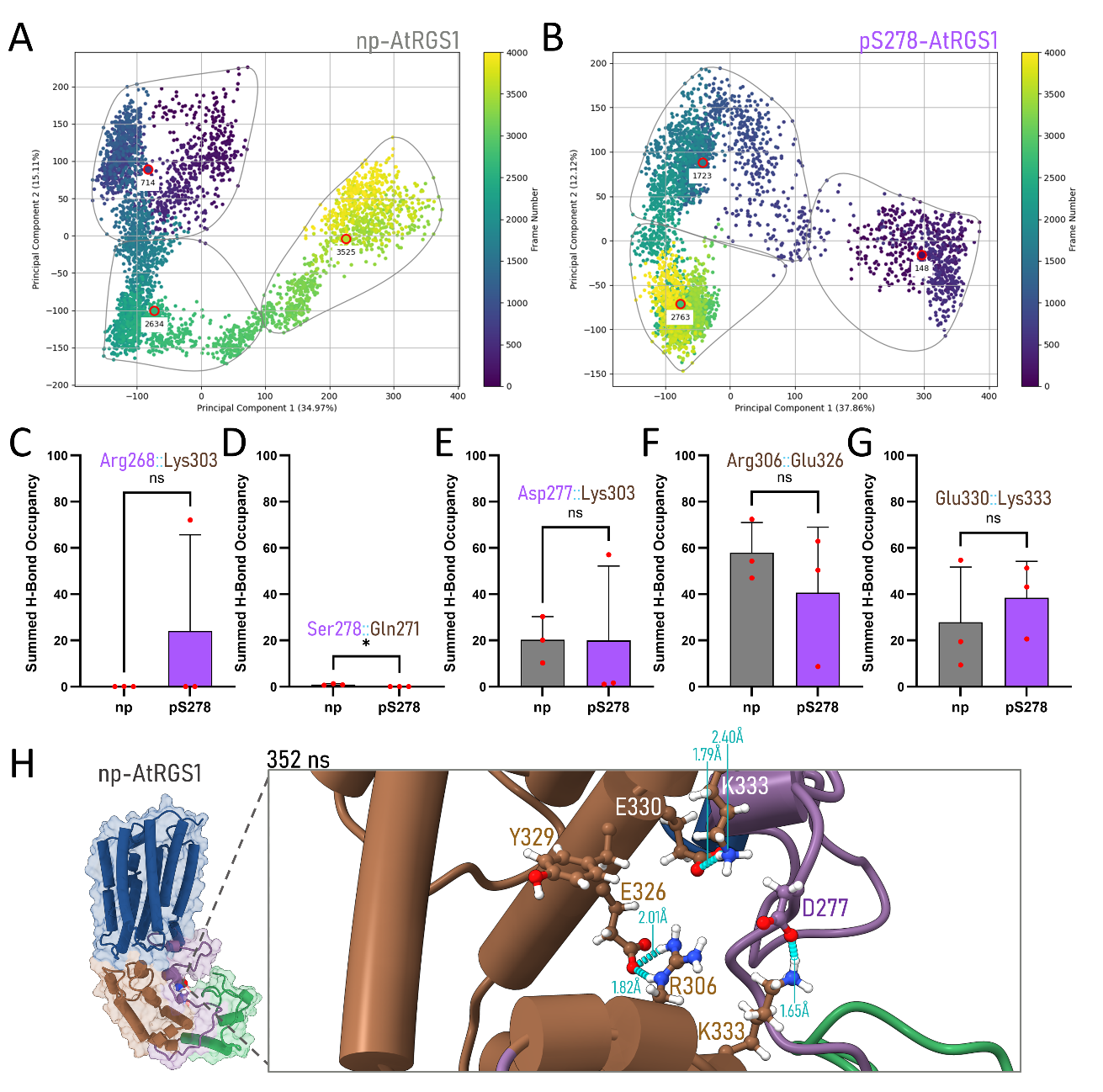
**

Principal component analyses (PCA) of representative MD production replicates were performed for **(A)** non-phosphorylated AtRGS1 (np-AtRGS1) and **(B)** phospho-Ser278-AtRGS1 (pS278-AtRGS1). Centroid frames, representing the dominant conformational states, are marked with red circles. **(C-G)** Quantification of the average hydrogen bond occupancy across independent replicates of both systems highlights the occupancy levels of previously identified residues in the centroid frames. Unpaired t-tests; *, p < 0.05, ns, non-significant. **(H)** A cartoon representation of the np-AtRGS1 representative frame at 352 ns illustrates the interactions and spatial proximity of the linker region with the regulatory domain of AtRGS1 in the non-phosphorylated system.

Supplementary Figure 4. Phosphonull mutants have increased stability compared to both wildtype- and phosphomimetic-complemented lines


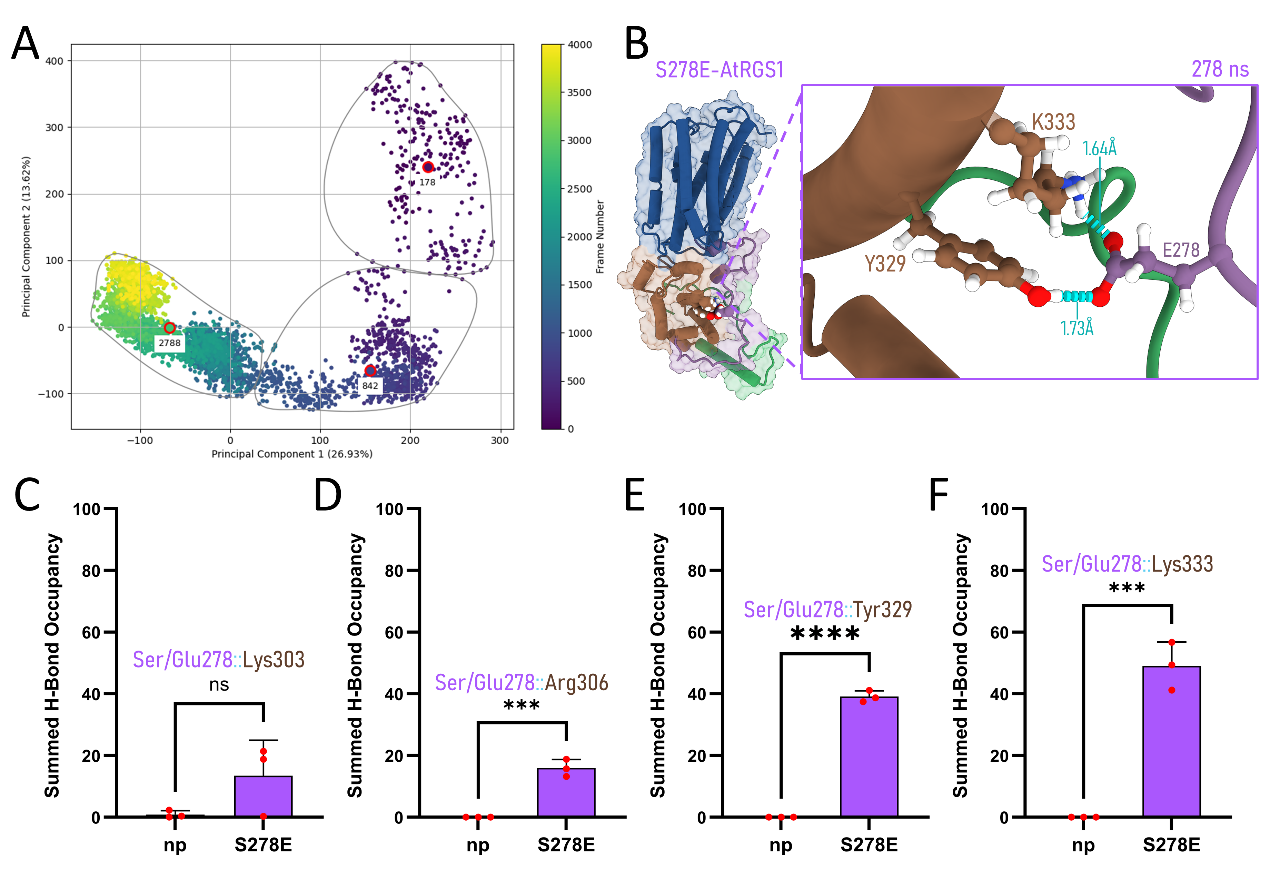


Principal component analysis (PCA) of representative MD production replicate of AtRGS1-S278E phosphomimetic system. Centroid frames, representing the dominant conformational states, are marked with red circles, and **(B)** the cartoon for the last cluster representative (278 ns) represented. (**C-F**) H-bond occupancy of Ser278 (np) or Glu278 mutant (S278E, purple) with RGS domain residues **(C)** K303, **(D)** R306, **(E)** Y329, and **(F)** K333. Unpaired t-tests; ***, p < 0.001, ****, p < 0.0001, ns, non-significant.

Supplementary Figure 5. RGS box domain conservation on non-viridiplantae eukaryotes.


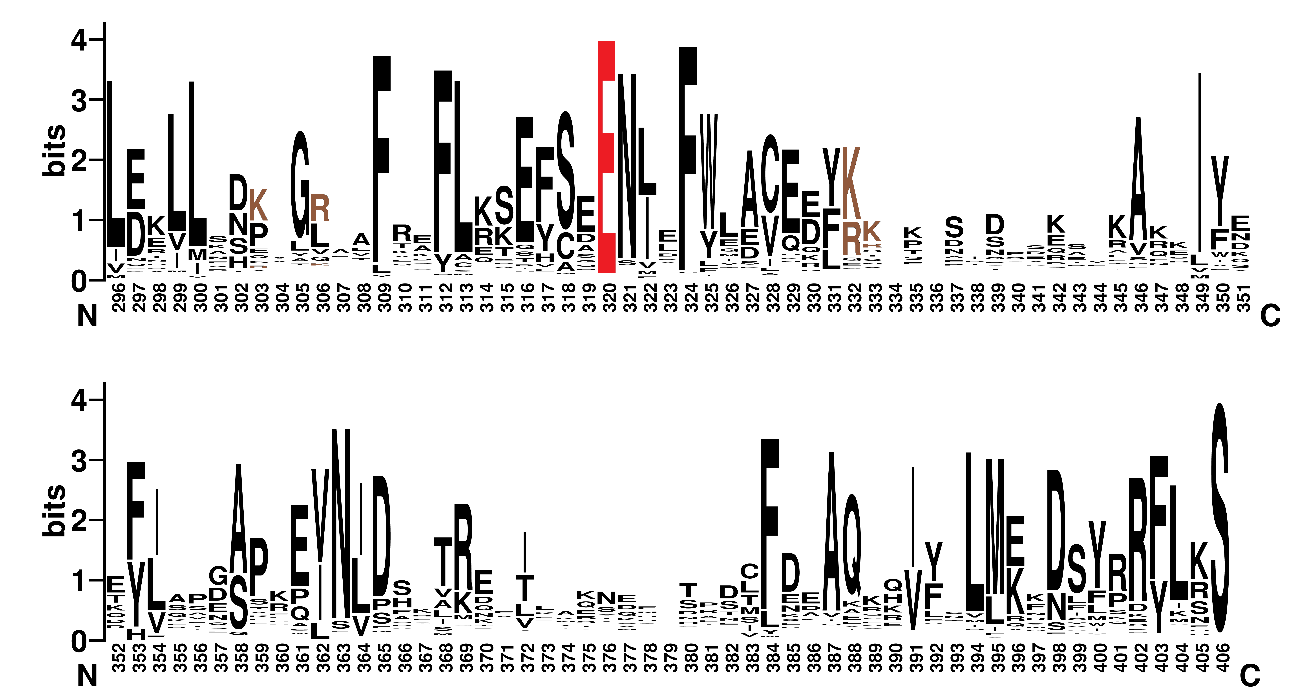


Sequence logo for the RGS box domain. A complete set of RGS-containing domains was retrieved and filtered to include only eukaryotic organisms, excluding the Viridiplantae group. Sequences were aligned to the Arabidopsis AtRGS1 domain and further filtered to create the sequence logo. Positive residues at the AtRGS1 linker-interacting positions (Lys303, Arg306, and Lys333) are shown in brown, while the universally conserved GAP-functional residue (Glu320) is highlighted in red.

Supplementary Figure 6. MD simulations of an evolutionary-distant 7TM-RGS protein show the same dynamics as for AtRGS1.


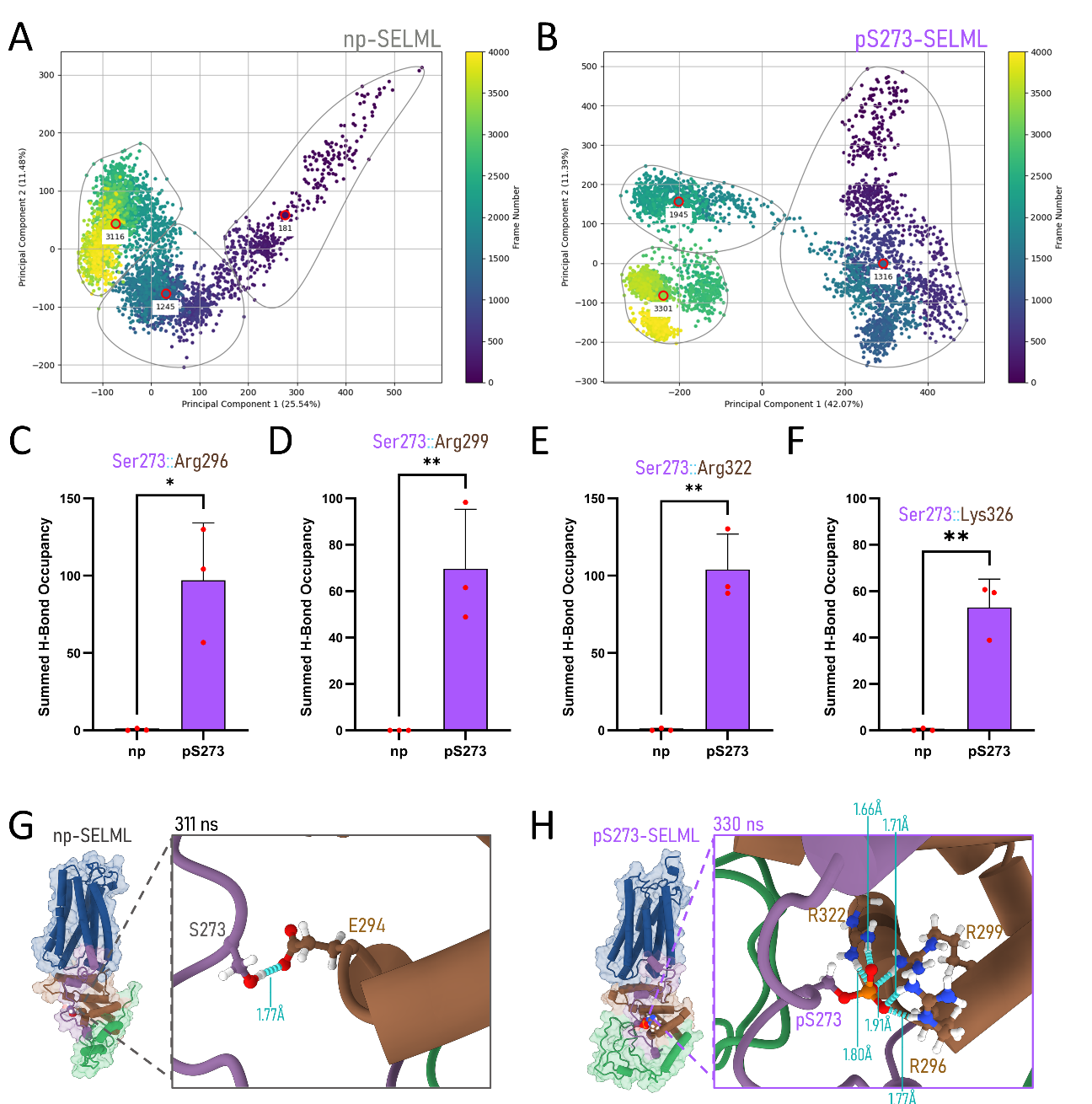


PCA was performed on representative molecular dynamics (MD) production replicates for (A) non-phosphorylated SELML (np-SELML) and (B) phospho-Ser273 SELML (pS273-SELML). Centroid frames, which represent the dominant conformational states, are marked with red circles. (C-F) Quantification of the average hydrogen bond occupancy across independent replicates for pS273 with RGS box residues equivalent to those in AtRGS1, including (C) Arg296, (D) Arg299, and (E) Lys326, corresponding to Lys303, Arg306, Tyr329, and Lys333 in the AtRGS1 sequence. Unpaired t-tests; *, p < 0.05, **, p < 0.01. The hydrogen bonds in the representative frames are depicted as cyan dotted lines, showing their respective distances for **(G)** np-S273 and **(H)** pS273.

Supplementary Figure 7. Confocal microscopy of etiolated Arabidopsis seedlings hypocotyl.


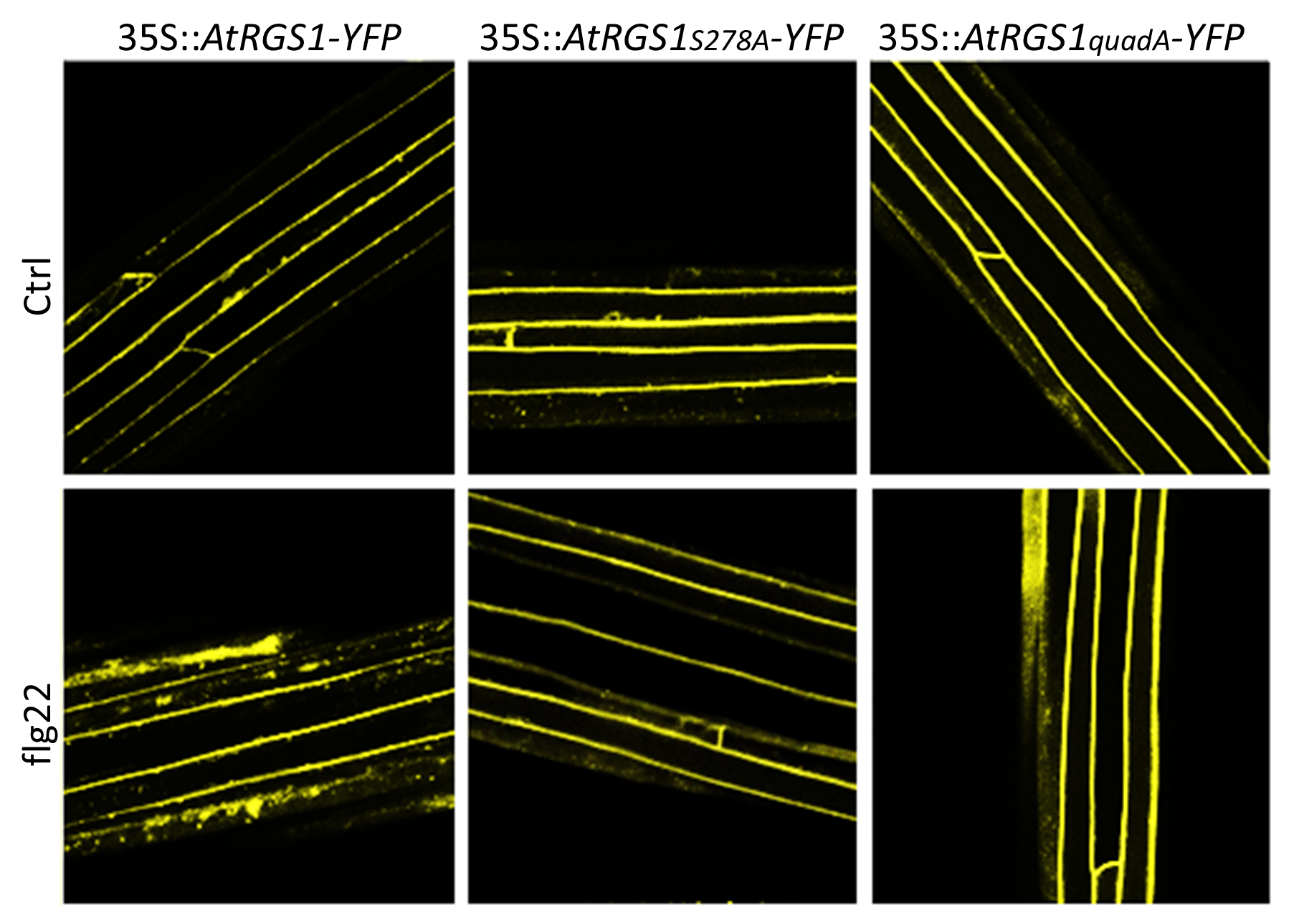


Representative confocal images are shown for Arabidopsis rgs1-2 seedlings complemented with wild-type, S278A, and quadA versions of AtRGS1 fused to YFP. The seedlings were treated with either water (Ctrl) or 100 nM flg22. Images illustrate the elongated hypocotyls after two days of dark treatment. Quantification of these results, including all replicates, is provided in the main text **(Figure 3D).**

Supplementary Figure 8. Wild type and phosphomutant AtRGS1 degradation kinetics. Loss of phosphorylation at S278 or within the C-tail stabilizes AtRGS1.

**
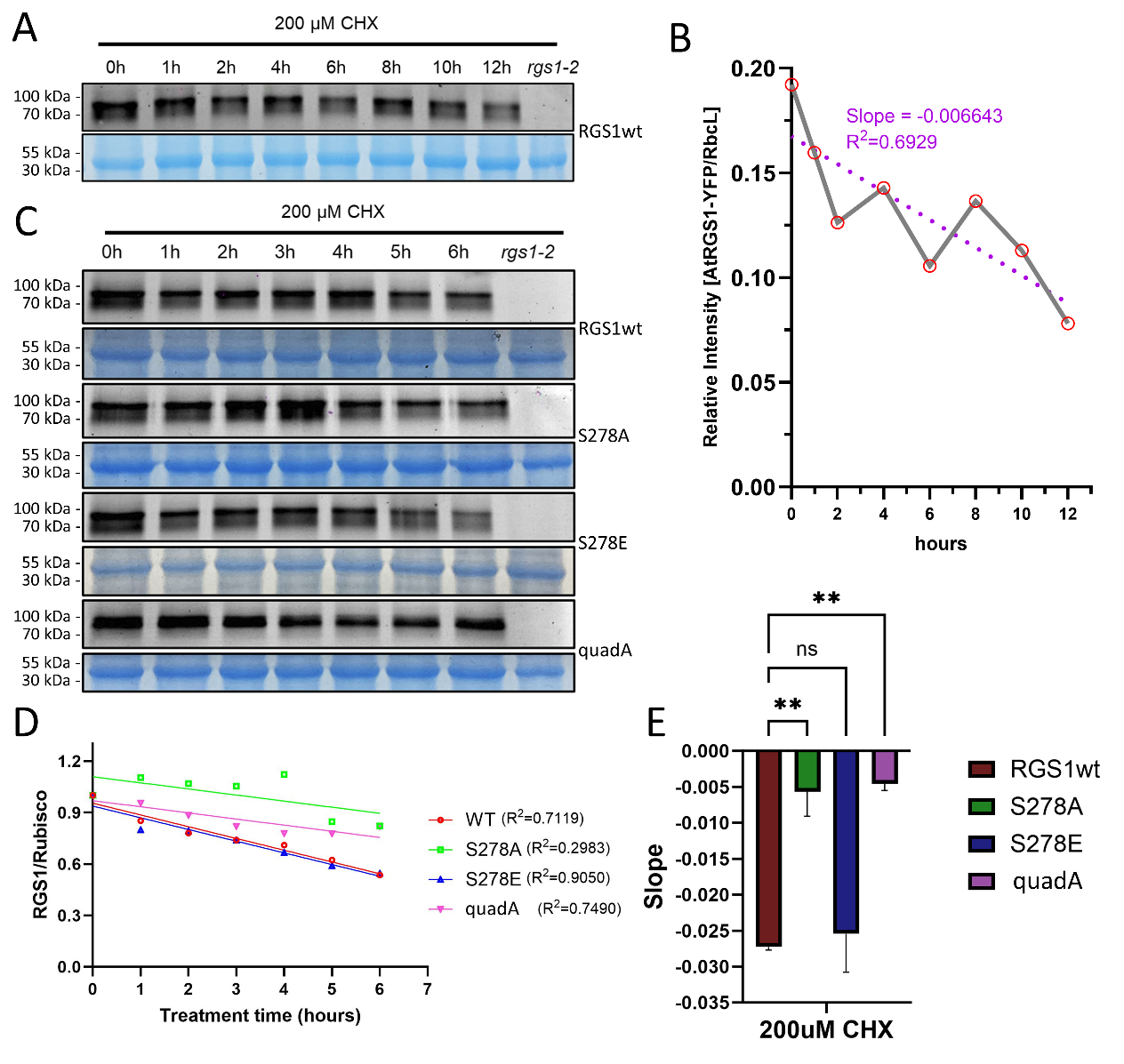
**

**(A)** Stability of the wild-type AtRGS1 complemented line was evaluated over 12 hours of cycloheximide (CHX) treatment. **(B)** AtRGS1-YFP protein levels were estimated from Western Blot data in panel A, with slope determined by linear fitting and shown with the R-squared value. Coomassie-stained gels show the Rubisco large subunit (RbcL, 52 kDa) as a total loading control, while blots indicate bands around 80 kDa corresponding to AtRGS1-YFP. Ladder upper and lower band sizes are indicated on the left. **(C)** Mutant impairment was observed for up to 6 hours of CHX treatment. Complete blots and stained membranes are shown in **Figure S9**. **(D)** Relative levels with R-squared values and **(E)** slope comparisons are presented. ANOVA, post-hoc Tukey's test; **, p < 0.01, ns, non-significant.

Supplementary Figure 9. Uncropped blots and gels from *rgs1-2* complemented lines

**
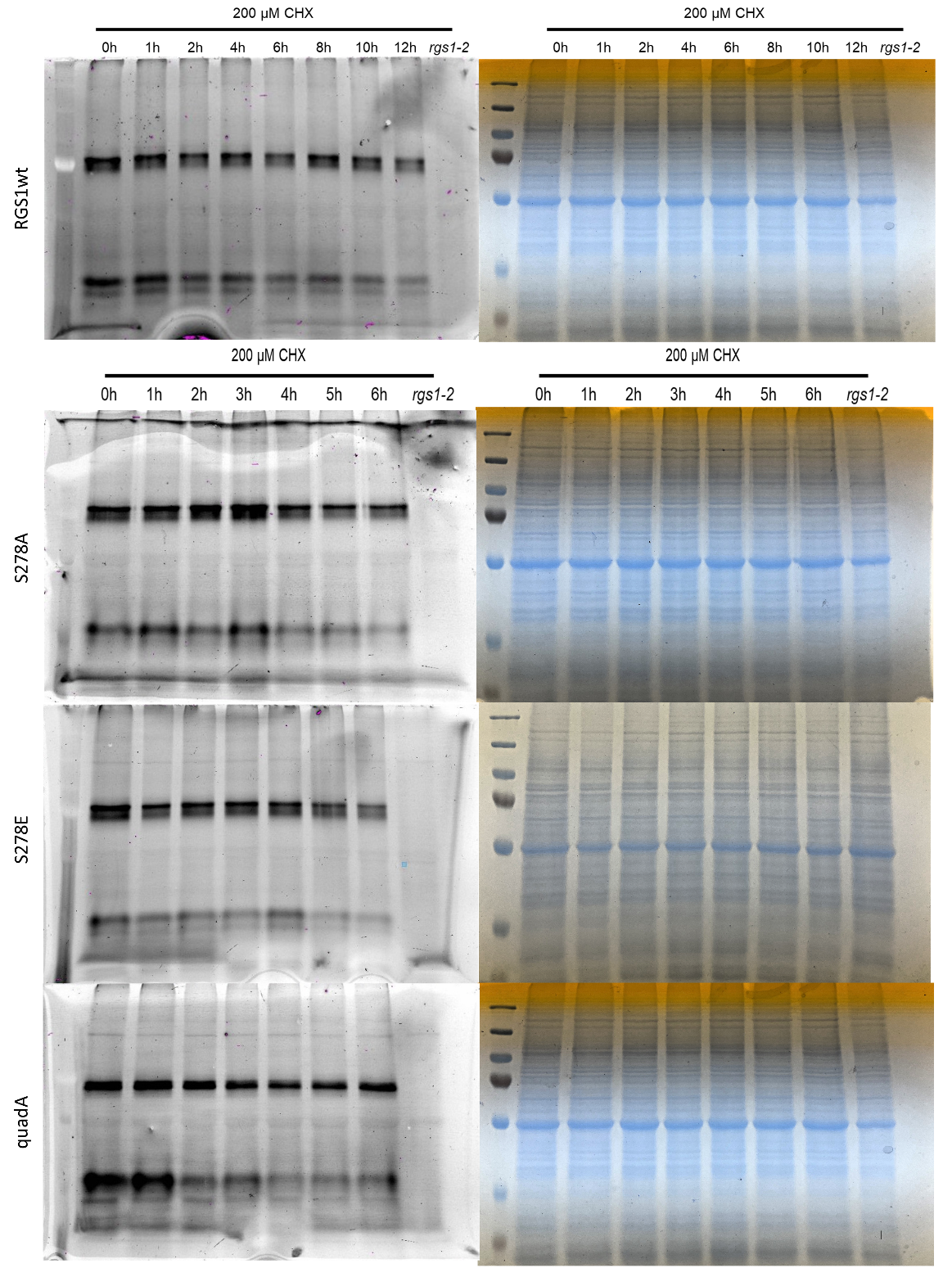
**

Uncropped blots of AtRGS1-YFP in *rgs1-2* complemented lines. Black and white images represent the YFP detected levels in the blotting membrane while colored images represent the comassie blue staining of the same membrane. Bands of interest and corresponding ladder sizes are shown in **Figure S8**.

Supplementary Figure 10. *In vivo* interaction of AtRGS1 phosphovariants with AtGPA1 phosphomimetic

**
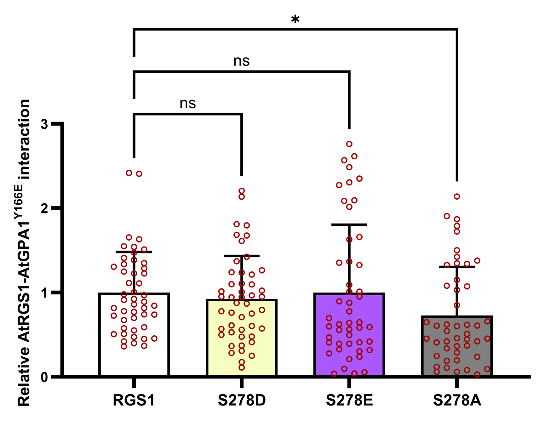
**

Quantification of the split-luciferase complementation assay in N. benthamiana leaves, assessing interactions between AtGPA1 Y166E and AtRGS1 mutants. Statistical analysis was performed using ANOVA followed by post-hoc Tukey's test, with comparisons made to the control (wild-type AtRGS1); *, p < 0.05, ns, non-significant.

Supplementary Figure 11. All-atom MD simulations on phosphorylated and non-phosphorylated AtRGS1 models bound to AtGPA1.

**
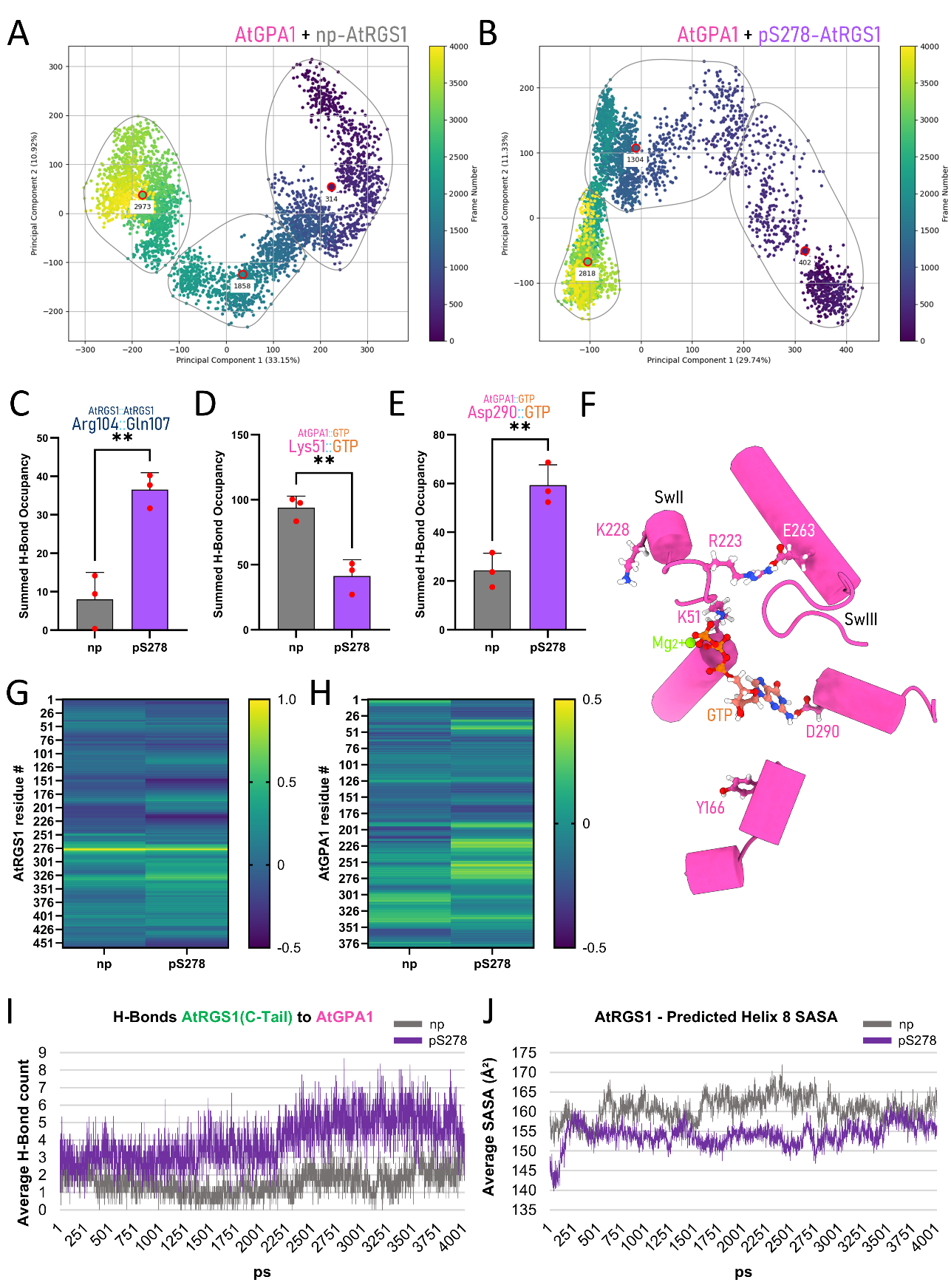
**

PCA was conducted on representative MD production replicates for **(A)** non-phosphorylated AtRGS1 bound to AtGPA1 (np-AtRGS1 + AtGPA1) and **(B)** phospho-Ser278-AtRGS1 bound to AtGPA1 (pS278-AtRGS1 + AtGPA1). Centroid frames, representing the dominant conformational states, are highlighted with red circles. **(C-E)** Quantification of the average hydrogen bond occupancy across independent replicates is shown for **(C)** AtRGS1 7TM residues (blue), **(D-E)** AtGPA1 residues (pink), and the GTP molecule (orange). Statistical significance was assessed using unpaired t-tests (*, p < 0.05; ns, non-significant). **(F)** A cartoon representation of the np-AtRGS1 + AtGPA1 representative frame at 297 ns illustrates the interactions and spatial proximity of AtGPA1-identified residues with the Switch II (SwII) and Switch III (SwIII) regions, which regulate GTP binding, exchange, and hydrolysis. **(G-H)** Average correlations of Ser278 with **(G)** other AtRGS1 residues and **(H)** AtGPA1 residues during the simulation replicates. **(I)** Average H-bond count per frame between AtRGS1 C-tail (407-459) and AtGPA1 residues. **(J)** Average Solvent Accessible Surface Area (SASA) per frame of the predicted helix 8 in AtRGS1 over 400 ns of the AtRGS1–AtGPA1 dimer simulations. The line plots show the average SASA for residues 253–262 across all replicates.

SUPPLEMENTARY TABLES

Supplementary Table I. Summed hydrogen bond (H-bond) occupancies for monomeric AtRGS1 RGS box residues interacting with TIP3 water molecules

| **TIP3-Paired Residue** | **AtRGS1-np Summed H-Bond Occupancy** | **STD** | **AtRGS1-pS278 Summed H-Bond Occupancy** | **STD** | **p-value** |
| --- | --- | --- | --- | --- | --- |
| **LYS303** | 93.887 | 9.562 | 106.510 | 17.202 | 0.329 |
| **ARG306** | 237.377 | 49.704 | 95.173 | 37.050 | 0.016 |
| **TYR329** | 66.547 | 5.516 | 29.793 | 15.734 | 0.019 |
| **LYS333** | 98.143 | 33.742 | 71.787 | 48.374 | 0.482 |

Supplementary Table II. Molecular dynamics system setup and parameters

| **System** | **PTMs** | **Disulfide bonds** | **pH** | **Exceptional Protonation** | **Lipid # (Upper/Lower)** | **Water Thick. (Å)** | **XY Init. Size (Å)** | **Net Charge** | **[NaCl] (mM)** | **Ion # (Na+/Cl-)** | **Eq. Method** | **Temp. (K)** |
| --- | --- | --- | --- | --- | --- | --- | --- | --- | --- | --- | --- | --- |
| AtRGS1 + AtGPA1 | GPA1: GLYM2/CYSP5 | AtRGS1: 5-11/84-153 | 7 | N.A. | SITO: 275/270, DUPC: 278/270, CER1: 55/54 | 35 | 185 | AtRGS1: +5, AtGPA1: -6 | 150 | 400/397 | NPT | 303.15 |
| AtRGS1-pS278 + AtGPA1 | AtRGS1: pS278, GPA1: GLYM2/CYSP5 | AtRGS1: 5-11/84-153 | 7 | RGS1-GLUP92 | SITO: 275/265, DUPC: 277/265, CER1: 55/53 | 35 | 185 | AtRGS1: +4, AtGPA1: -6 | 150 | 377/373 | NPT | 303.15 |
| AtRGS1 | N.A. | AtRGS1: 5-11/84-153 | 7 | N.A. | SITO: 155/155, DUPC: 156/155, CER1: 31/31 | 35 | 140 | AtRGS1: +5 | 150 | 205/210 | NPT | 303.15 |
| AtRGS1-pS278 | AtRGS1: pS278 | AtRGS1: 5-11/84-153 | 7 | N.A. | SITO: 155/155, DUPC: 155/155, CER1: 31/31 | 35 | 140 | AtRGS1: +3 | 150 | 211/214 | NPT | 303.15 |
| AtRGS1-S278E | N.A. | AtRGS1: 5-11/84-153 | 7 | N.A. | SITO: 155/155, DUPC: 156/155, CER1: 31/31 | 35 | 140 | AtRGS1: +4 | 150 | 208/212 | NPT | 303.15 |
| SELML | N.A. | SELML: 4-10/83-154 | 7 | N.A. | SITO: 155/155, DUPC: 156/155, CER1: 31/31 | 35 | 140 | SELML: +4 | 150 | 239/243 | NPT | 303.15 |
| SELML-pS273 | SELML: pS273 | SELML: 4-10/83-154 | 7 | N.A. | SITO: 155/155, DUPC: 156/155, CER1: 31/31 | 35 | 140 | SELML: +2 | 150 | 226/228 | NPT | 303.15 |

REFERENCES

[1] Katoh K, Standley DM. MAFFT Multiple Sequence Alignment Software Version 7: Improvements in Performance and Usability. Mol Biol Evol 2013;30:772–80. https://doi.org/10.1093/MOLBEV/MST010.

[2] Minh BQ, Schmidt HA, Chernomor O, Schrempf D, Woodhams MD, von Haeseler A, et al. IQ-TREE 2: New Models and Efficient Methods for Phylogenetic Inference in the Genomic Era. Mol Biol Evol 2020;37:1530–4. https://doi.org/10.1093/molbev/msaa015.

[3] Letunic I, Bork P. Interactive Tree Of Life (iTOL) v5: an online tool for phylogenetic tree display and annotation. Nucleic Acids Res 2021;49:W293–6. https://doi.org/10.1093/NAR/GKAB301.

[4] Li W, Godzik A. Cd-hit: a fast program for clustering and comparing large sets of protein or nucleotide sequences. Bioinformatics 2006;22:1658–9. https://doi.org/10.1093/bioinformatics/btl158.

[5] Crooks GE, Hon G, Chandonia JM, Brenner SE. WebLogo: a sequence logo generator. Genome Res 2004;14:1188–90. https://doi.org/10.1101/GR.849004.

[6] Abramson J, Adler J, Dunger J, Evans R, Green T, Pritzel A, et al. Accurate structure prediction of biomolecular interactions with AlphaFold 3. Nature 2024 630:8016 2024;630:493–500. https://doi.org/10.1038/s41586-024-07487-w.

[7] Adjobo-Hermans MJW, Goedhart J, Gadella TWJ. Plant G protein heterotrimers require dual lipidation motifs of Gα and Gγ and do not dissociate upon activation. J Cell Sci 2006;119:5087–97. https://doi.org/10.1242/jcs.03284.

[8] Jumper J, Evans R, Pritzel A, Green T, Figurnov M, Ronneberger O, et al. Highly accurate protein structure prediction with AlphaFold. Nature 2021 596:7873 2021;596:583–9. https://doi.org/10.1038/s41586-021-03819-2.

[9] Wu EL, Cheng X, Jo S, Rui H, Song KC, Dávila-Contreras EM, et al. CHARMM-GUI Membrane Builder toward realistic biological membrane simulations. J Comput Chem 2014;35:1997–2004. https://doi.org/10.1002/JCC.23702.

[10] Lomize AL, Todd SC, Pogozheva ID. Spatial arrangement of proteins in planar and curved membranes by PPM 3.0. Protein Science 2022;31:209–20. https://doi.org/10.1002/PRO.4219.

[11] Emami S, Azadmard-Damirchi S, Peighambardoust SH, Hesari J, Valizadeh H, Faller R. Molecular dynamics simulations of ternary lipid bilayers containing plant sterol and glucosylceramide. Chem Phys Lipids 2017;203:24–32. https://doi.org/10.1016/j.chemphyslip.2017.01.003.

[12] Jo S, Kim T, Iyer VG, Im W. CHARMM-GUI: A web-based graphical user interface for CHARMM. J Comput Chem 2008;29:1859–65. https://doi.org/10.1002/JCC.20945.

[13] Abraham MJ, Murtola T, Schulz R, Páll S, Smith JC, Hess B, et al. GROMACS: High performance molecular simulations through multi-level parallelism from laptops to supercomputers. SoftwareX 2015;1–2:19–25. https://doi.org/10.1016/J.SOFTX.2015.06.001.

[14] Hopkins CW, Le Grand S, Walker RC, Roitberg AE. Long-time-step molecular dynamics through hydrogen mass repartitioning. J Chem Theory Comput 2015;11:1864–74. https://doi.org/10.1021/CT5010406/ASSET/IMAGES/MEDIUM/CT-2014-010406_0018.GIF.

[15] Brown DK, Penkler DL, Sheik Amamuddy O, Ross C, Atilgan AR, Atilgan C, et al. MD-TASK: a software suite for analyzing molecular dynamics trajectories. Bioinformatics 2017;33:2768–71. https://doi.org/10.1093/bioinformatics/btx349.

[16] Humphrey W, Dalke A, Schulten K. VMD: Visual molecular dynamics. J Mol Graph 1996;14:33–8. https://doi.org/10.1016/0263-7855(96)00018-5.

[17] Mitternacht S. FreeSASA: An open source C library for solvent accessible surface area calculations. F1000Res 2016;5:189. https://doi.org/10.12688/f1000research.7931.1.

[18] Pettersen EF, Goddard TD, Huang CC, Meng EC, Couch GS, Croll TI, et al. <scp>UCSF ChimeraX</scp> : Structure visualization for researchers, educators, and developers. Protein Science 2021;30:70–82. https://doi.org/10.1002/pro.3943.

[19] Watkins JM, Ross-Elliott TJ, Shan X, Lou F, Dreyer B, Tunc-Ozdemir M, et al. Differential regulation of G protein signaling in Arabidopsis through two distinct pathways that internalize AtRGS1. Sci Signal 2021;14. https://doi.org/10.1126/SCISIGNAL.ABE4090/SUPPL_FILE/SCISIGNAL.ABE4090_SM.PDF.

[20] Zhang X, Henriques R, Lin SS, Niu QW, Chua NH. Agrobacterium-mediated transformation of Arabidopsis thaliana using the floral dip method. Nat Protoc 2006;1:641–6. https://doi.org/10.1038/NPROT.2006.97.

[21] Urano D, Phan N, Jones JC, Yang J, Huang J, Grigston J, et al. Endocytosis of Seven-Transmembrane RGS Protein Activates G- protein Coupled Signaling in Arabidopsis. Nat Cell Biol 2012;14:1079. https://doi.org/10.1038/NCB2568.

[22] Simoni EB, Oliveira CC. The Split-Luciferase Complementation Assay to Detect and Quantify Protein-Protein Interactions in Planta. Methods Mol Biol 2024;2724:247–55. https://doi.org/10.1007/978-1-0716-3485-1_18.

[23] Liang X, Ma M, Zhou Z, Wang J, Yang X, Rao S, et al. Ligand-triggered de-repression of Arabidopsis heterotrimeric G proteins coupled to immune receptor kinases. Cell Research 2018 28:5 2018;28:529–43. https://doi.org/10.1038/s41422-018-0027-5.
